# The Effects of Transcranial Magnetic Stimulation on Alcohol Craving, Efficacy, Emotion, and Cognitive Function in Patients With Alcohol Use Disorders: A Systematic Review and Meta‐Analysis of Randomized Controlled Trial

**DOI:** 10.1002/brb3.71605

**Published:** 2026-07-26

**Authors:** Jiayuan Song, Xingchen He, Ji Ouyang, Zhaoying Li, Yanlin liu, Han Yu, Aimin Li, Hongwei Liu, Haixia Fan, Fan Yao

**Affiliations:** ^1^ College of Integrative Medicine Changchun University of Chinese Medicine Changchun China; ^2^ The Affiliated Guangzhou Hospital of TCM of Guangzhou University of Chinese Medicine Guangzhou China; ^3^ College of Traditional Chinese medicine Hunan University of Traditional Chinese Medicine Changsha Hunan China; ^4^ School of Acupuncture and Massage Changchun University of Traditional Chinese Medicine Changchun China; ^5^ Department of Neurology, Taiyuan City Central Hospital The Ninth Clinical Medical College of Shanxi Medical University Taiyuan Shanxi China; ^6^ Department of Sleep center First Hospital of Shanxi Medical University Taiyuan Shanxi China; ^7^ Preventive Treatment of Disease Department Changchun University of Traditional Chinese Medicine Changchun China

**Keywords:** alcohol craving, alcohol use disorder, meta‐analysis, transcranial magnetic stimulation

## Abstract

**Objectives:**

This study aimed to evaluate the effects of transcranial magnetic stimulation (TMS) on alcohol craving, abstinence days, alcohol intake, anxiety, depression, and cognitive function in patients with alcohol use disorder (AUD), providing evidence to support precision treatment strategies.

**Methods:**

As of March 6, 2026, we have systematically searched eight databases (PubMed, EMBASE, Cochrane Library, Web of Science, China National Knowledge Infrastructure, China Science Journal Database, Wanfang Database, and China Biomedical Literature Service System). The Cochrane Risk of Bias Tool for Randomized Trials (RoB 2) was used to assess the quality of the included randomized controlled trial (RCT) studies. Stata15 was employed for the meta‐analysis.

**Results:**

We included 19 RCT studies involving 942 patients with AUD. Compared with the sham stimulation group, TMS significantly reduced alcohol craving in patients with AUD. Subgroup analysis showed significant effect sizes in cases where the TMS type was continuous theta burst stimulation (cTBS), the number of sessions was 10 or 15, the frequency was 10 Hz, and the follow‐up duration was ≤ 1 month or ≥ 3 months. Meta‐regression indicated that disease type might be the main reason for the high heterogeneity in alcohol craving. Compared with the sham stimulation group, TMS showed significant differences in maintaining abstinence days, reducing alcohol intake, alleviating depressive symptoms, and improving cognitive function.

**Conclusions:**

Current evidence indicates that TMS can effectively alleviate craving in patients with AUD and demonstrate significant advantages in maintaining abstinence days, reducing alcohol intake, alleviating depressive symptoms, and improving cognitive function. Given the high degree of heterogeneity, the results were interpreted with caution. In the future, more large‐sample, multi‐center, double‐blind RCT will be needed to supplement and validate the results of this study.

## Abstract 2

This meta‐analysis of 19 randomized controlled trials (N = 942 patients with alcohol use disorder) evaluated the therapeutic effects of transcranial magnetic stimulation (TMS). Compared with sham stimulation, TMS significantly reduced alcohol craving, with notable subgroup effects for continuous theta burst stimulation (cTBS), 10 Hz frequency, and 10 or 15 sessions. Benefits were sustained at follow‐ups ≤1 month and ≥3 months. TMS also significantly increased abstinence days, reduced alcohol intake, alleviated depressive symptoms, and improved cognitive function. Meta‐regression suggested that disease type (e.g., comorbid psychiatric conditions) may contribute to heterogeneity. These findings support TMS as a promising non‐invasive intervention for AUD, though further large‐scale, multicenter, double‐blind RCTs are warranted to confirm results.

## 1 Introduction

Alcohol use disorder (AUD) is a severe and widespread mental disorder, characterized primarily by compulsive heavy drinking and an inability to control drinking behavior (American Psychiatric Association 1980, 2013). It is one of the leading causes of global disease burden and premature death (Danpanichkul et al. 2025; Rehm et al. 2017). Data from the Global Burden of Disease 2021 showed that AUD increased by 14.66% from 2000 to 2021 (Danpanichkul et al. 2025). A retrospective cohort study with a 12‐year follow‐up revealed that individuals with AUD have a life expectancy 21–27 years shorter than that of the general population (Kážmér et al. 2025). Moreover, due to its chronic and recurrent nature, AUD has a close causal relationship with many life‐threatening diseases, such as sleep disorders, liver disease, cardiovascular disease, cerebellar damage, and cancer (García‐Dolores et al. 2025; Parry et al. 2011; Yu et al. 2025). Despite this, the proportion of patients with AUD seeking treatment is significantly low. Globally, only one in six patients with AUD receive treatment (Mekonen et al. 2021); in the United States, 94% of patients with AUD do not receive medication or counseling (Mintz et al. 2021); in South Korea, only 2.6% of patients with AUD seek mental health services (Rim et al. 2023). Therefore, finding effective, acceptable, and low‐recurrence treatment methods for patients with AUD has become an urgent public health issue.

Currently, the treatment methods for patients with AUD mainly include medication and psychological therapies such as cognitive‐behavioral therapy or motivational interviewing (Johansson et al. 2021). Although progress has been made with these approaches, their clinical utility is often limited not only by medication‐related side effects but also by issues of accessibility and affordability. Many patients face barriers to receiving these treatments due to limited availability of specialized services and the high costs associated with sustained care. New treatment methods are needed to address issues such as alcohol craving, withdrawal effects, and consumption in patients with AUD, as well as commonly comorbid conditions such as anxiety, depression, and cognitive impairment (Ummels et al. 2022; G. Wang et al. 2023; Yan et al. 2025).

Transcranial magnetic stimulation (TMS), as an emerging non‐invasive brain stimulation technique, is based on the principle of electromagnetic induction in the brain's electric field. It aims to regulate the excitability and plasticity of the target cortex and exerts a broader impact on the network in this region (Jannati et al. 2023; Wagner et al. 2007). In terms of safety and feasibility, TMS is considered safe and feasible when applied in accordance with the recommended safety and application guidelines endorsed by the International Federation of Clinical Neurophysiology (Rossi et al. 2021; Rossini et al. 2015). A recently published meta‐analysis (Treiber et al. 2025) showed that TMS reduced alcohol craving in patients with AUD, with effects lasting for at least 3 months; stimulation targeting the medial prefrontal cortex demonstrated better efficacy. However, this study did not conduct further subgroup analysis based on sample size, stimulus parameters (stimulus form, frequency, intensity, and number of sessions), methods for assessing alcohol craving, AUD patient type, country, and age. In addition, a meta‐analysis conducted by Kim et al. (2025) showed that TMS had no significant intervention effect on alcohol craving in patients with AUD. There are critical evidence gaps in providing a comprehensive and optimal TMS protocol for patients with AUD while focusing on withdrawal effects, alcohol consumption, mood, and cognition. Therefore, this study aims to comprehensively evaluate the impact of TMS on alcohol craving, withdrawal effects, alcohol intake, emotion, and cognitive function in patients with AUD by including randomized controlled trials (RCTs) through systematic review and meta‐analysis so as to provide an evidence base for promoting the precision of treatment regimens.

## 2 Methods

### 2.1 Protocol and Registration

This study reports the included original studies in accordance with the Preferred Reporting Items for Systematic Reviews and Meta‐Analyses (PRISMA) (Moher et al. 2009) (Table S1) and has been registered in the PROSPERO database (CRD420251091394).

### 2.2 Search Strategy

We conducted a search of the following eight databases on March 6, 2026 (PubMed, EMBASE, Cochrane Library, Web of Science, China National Knowledge Infrastructure, China Science Journal Database, Wanfang Database, and China Biomedical Literature Service System). The search was limited to English or Chinese languages. The search terms used were “blood alcohol content,” “alcoholic intoxication,” “alcoholism,” “drinking behavior,” “alcohol abuse,” “alcohol use,” “transcranial Magnetic Stimulation,” “noninvasive brain stimulation,” “TMS.” The detailed search strategy is shown in (Table S2). In addition, we also searched the reference lists of eligible articles to identify potential studies that might be included.

### 2.3 Inclusion Criteria

Based on the following PICOS criteria, the results are as follows:

1. Study subjects: Adult patients diagnosed with AUD, alcohol dependence (AD), or alcohol abuse; diagnoses are based on the Diagnostic and Statistical Manual of Mental Disorders (DSM‐IV/V), the International Classification of Diseases 10 (ICD‐10), the Mini International Neuropsychiatric Interview (Mini), or other clinical diagnoses.
2. Interventions: TMS is applied to patients with AUD; detailed information such as the type of TMS, stimulation target, equipment, coil, location, intensity, frequency, and duration is reported.
3. Control: Receiving sham‐TMS.
4. Outcomes: The primary outcome is alcohol craving; the secondary outcomes include abstinence days, alcohol intake, anxiety, depression, and cognitive function. Alcohol craving was selected as the primary outcome because it is a core clinical feature of AUD and a key driver of relapse. In addition, craving was the most consistently reported outcome across the included RCTs, allowing for a larger pooled sample size and more robust meta‐analytic estimates. Compared with alcohol consumption and abstinence‐related measures, craving is considered a more proximal and sensitive indicator of treatment response to neuromodulation interventions such as TMS.
5. Study type: RCT.

### 2.4 Exclusion Criteria

### Exclusion criteria are as follows:

1. studies in which the study subjects have comorbid diseases that may affect the judgment of results;
2. studies combining other neuromodulation techniques (such as transcranial direct current stimulation);
3. unable to obtain the full text;
4. study type: Non‐RCT; and
5. studies where data cannot be extracted or are incomplete.

### 2.5 Selection Process

All studies were imported into Endnote (Version 21.0) for management and removal of duplicate studies. Two well‐trained researchers independently screened the titles and abstracts to identify studies that might meet the criteria for full‐text evaluation. The full texts were further obtained, and the initially eligible studies were screened to determine the final included studies. If there was a disagreement between the two researchers during the entire screening process, a decision would be made after discussion with a third researcher.

### 2.6 Data Extraction

In accordance with the PRISMA guidelines, two researchers systematically recorded study characteristics (authors, year, country, and sample size), characteristics of study subjects (age and diagnostic criteria for AUD), intervention parameters (TMS type, frequency, intensity, number of sessions, total pulses, time, target, and follow‐up duration), control group (sham‐TMS), and outcome indicators (alcohol craving, abstinence days, alcohol intake, anxiety, depression, and cognitive function). In case of disputes, a decision will be made after discussion with a third researcher.

### 2.7 Quality Assessment

The quality assessment process was independently conducted by two researchers to evaluate the quality of studies and the rigor of methodology. The Cochrane Risk of Bias Tool for Randomized Trials (RoB 2) (Sterne et al. 2019) was used, which assesses the likelihood of bias in the following five domains: (1) bias arising from the randomization process; (2) bias arising from deviations from the intended interventions; (3) bias due to missing outcome data; (4) bias in outcome measurement; (5) bias in outcome reporting. Finally, RCTs were classified into “good quality,” “moderate quality,” or “poor quality.” In case of disputes, a decision was made after discussion with a third researcher.

### 2.8 Statistical Analyses

Meta‐analysis was performed using Stata15. All outcome indicators in this study are continuous variables. Therefore, the mean difference (MD) was used for continuous variables with unified evaluation criteria, and the standardized mean difference (SMD) was used for continuous variables with different evaluation criteria to express the combined statistics, with their 95% confidence intervals calculated. Heterogeneity was evaluated using *I* ^2^ and the *p*‐value of the Cochrane *Q* statistic. If *I* ^2^ ≥ 50% and *p* < 0.1, it indicates significant heterogeneity, and a random‐effects model was used; if *I* ^2^ < 50% and *p* ≥ 0.1, it indicates low heterogeneity, and a fixed‐effects model was used. Sources of heterogeneity were explored through meta‐regression analysis and subgroup analysis. Sensitivity analysis was conducted to assess the robustness of the evaluation results by excluding studies one by one. When the number of studies was > 10, Egger's test was used to evaluate publication bias. A *p*‐value < 0.05 was considered statistically significant.

## 3 Results

### 3.1 Literature Screening Process

**FIGURE 1:**
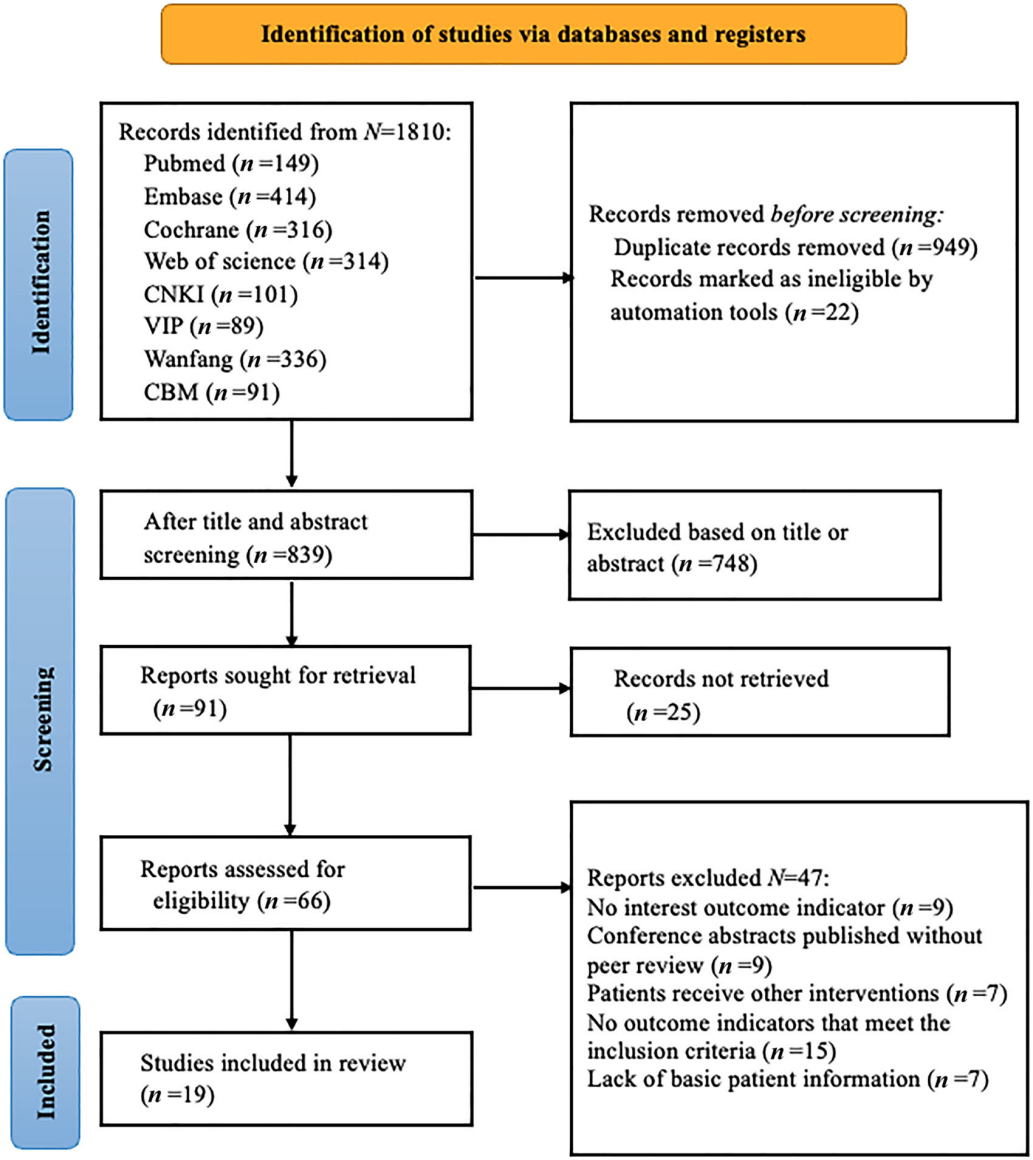
Literature screening process.

We searched eight databases and identified a total of 1810 records. Among them, 949 were duplicates and 22 were recorded as ineligible by automated tools. We then screened the titles and abstracts of 839 records. After excluding 748 records, we reviewed the full texts of 66 records. Finally, 19 records met the inclusion criteria (Addolorato et al. 2017; Belgers et al. 2022; Ceccanti et al. 2015; Durazzo et al. 2025; Fang et al. 2022; Harel et al. 2022; Herremans et al., 2012, 2015, 2013; Hoven et al. 2023; Hu et al. 2022; Jansen et al. 2019; McCalley et al. 2023; Mishra et al. 2010; Padula et al. 2024; Perini et al. 2020; Raikwar et al. 2020; H. Wang et al., 2025; Y. Wang et al., 2025). See Figure 1.

### 3.2 Study Characteristics

A total of 19 studies involving 902 patients with AUD were included. All studies were RCTs. Among them, six studies used high‐frequency repetitive transcranial magnetic stimulation (HrTMS), seven studies used repetitive transcranial magnetic stimulation (rTMS), two studies used deep transcranial magnetic stimulation (dTMS), three studies used intermittent theta burst stimulation (iTBS), and one study used continuous theta burst stimulation (cTBS). In terms of stimulation targets, nine studies applied TMS to the right dorsolateral prefrontal cortices (rDLPFC), six studies applied TMS to the left dorsolateral prefrontal cortices (lDLPFC), one study applied TMS to the dorsolateral prefrontal cortices bilateral (DLPFC bilateral), and three studies applied TMS to the medial prefrontal cortex (mPFC). Table 1 describes the baseline characteristics of the included study populations. Table 2 reports TMS‐related parameters in detail.

**TABLE 1:** Characteristics of studies included.

First author	Publication year	Study type	Country/region	Age (years) at baseline	Sample size	Participants for analysis	Diagnostic criteria	Interventions	Outcome	Assessment time points
Mishra	2010	Single‐blind, sham‐controlled, RCT	India	38.97 ± 8.23	45	AD	ICD‐10	G1: HrTMS G2: sham	ACQ‐ NOW	Pre, post, FU‐1M
Herremans	2012	Single‐blind, sham‐controlled, RCT	Belgium	49 ± 9.96	36	AD	NR	G1: HrTMS G2: sham	OCDS	Pre, post
Herremans	2013	Single‐blind, sham‐controlled, RCT	Belgium	48.14 ± 9.32	29	AD	Mini	G1: HrTMS G2: sham	OCDS	Pre, post
Herremans	2015	Single‐blind, sham‐controlled, RCT	Belgium	45.2 ± 9.3	33	AD	Mini	G1: HrTMS G2: sham	AUQ, OCDS	Pre, post
Ceccanti	2015	Double ‐blind, sham‐controlled, RCT	Italy	45.0 ± 11.07	18	AD	DSM‐IV	G1: dTMS G2: sham	VAS, alcohol intake	Pre, post, FU‐1 M, FU‐2M
Addolorato	2017	RCT	Italy	48.6 ± 9.9	14	AUD	DSM‐V	G1: rTMS G2: sham	OCDS, STAI, Zung, Abstinence days, alcohol intake	Pre, post, FU‐1M
Jansen	2019	Single‐blind, sham‐controlled, RCT	Netherlands	42.65 ± 9.78	36	AUD	NR	G1: rTMS G2: sham	AUQ	Pre, post
Perini	2020	Double ‐blind, sham‐controlled, RCT	Sweden	52.01 ± 9.11	45	AD	DSM‐IV	G1: rTMS G2: sham	AUQ, PACS	Pre, post, FU‐1 W, FU‐2 W, FU‐4 W, FU‐8 W, FU‐12W
Raikwar	2020	Single‐blind, sham‐controlled, RCT	India	37.13 ± 7.07	60	AD	ICD‐10	G1: rTMS G2: sham	ACQ‐ NOW	Pre, post, FU‐2W
Feng	2022	Single‐blind, sham‐controlled, RCT	China	37.93 ± 5.87	72	AUD	DSM‐V	G1: HrTMS G2: sham	MoCA	Pre, post, FU‐14D
Harel	2022	Double ‐blind, sham‐controlled, RCT	Israel	43.1 ± 9.18	51	AD	DSM‐V	G1: dTMS G2: sham	PACS, alcohol intake, CPRS, BDI	Pre, post, FU‐1 W, FU‐2 W, FU‐4 W, FU‐8 W, FU‐12W
Hu	2022	Double ‐blind, sham‐controlled, RCT	China	46.67 ± 10.99	144	AD	DSM‐IV	G1: rTMS G2: sham	OCDS, GAD‐7, MoCA	Pre, post, FU‐2 W, FU‐8 W, FU‐12 W, FU‐24W
Belgers	2022	Single‐blind, sham‐controlled, RCT	Netherlands	47.4 ± 8.9	34	AUD	DSM‐V	G1: rTMS G2: sham	VAS, AUQ, Abstinence days, alcohol intake	Pre, post, FU‐1 M, FU‐3 M, FU‐12M
McCalley	2023	Double ‐blind, sham‐controlled, RCT	America	45.9 ± 11.7	50	AUD	NR	G1: cTBS G2: sham	OCDS, AUQ, STAI, Abstinence days	Pre, post, FU‐1 M, FU‐2 M, FU‐3M
Hoven	2023	Single‐blind, sham‐controlled, RCT	Netherlands	44.35 ± 10.69	82	AUD	DSM‐IV	G1: HrTMS G2: sham	OCDS, AUQ, Abstinence days	Pre, post, FU‐3 M, FU‐6 M, FU‐12M
Padula	2024	Double ‐blind, sham‐controlled, RCT	America	44.4 ± 11.8	17	AUD	DSM‐V	G1: iTBS G2: sham	OCDS, Abstinence days, MASQ	Pre, post
Durazzo	2025	RCT	America	50.95 ± 13.94	44	AUD	DSM‐V	G1: iTBS G2: sham	Abstinence days	Pre, post, FU‐6M
Wang	2025	Double ‐blind, sham‐controlled, RCT	China	38.44 ± 7.76	52	AD	ICD‐10	G1: iTBS G2: sham	VAS, BAI, BDI	Pre, post, FU‐2W
Wang	2025	RCT	China	39.87 ± 0.12	80	AD	DSM‐V	G1: rTMS G2: sham	HAMA, HAMD, MMSE	Pre, FU‐4W

Abbreviations: AD, alcohol dependence; ACQ, the Acute Urge Questionnaire; ACQ‐NOW, the Alcohol Craving Questionnaires; AUD, alcohol use disorder; BDI, Beck Depression Inventory; CPRS, Comprehensive Psychopathological Rating Scale; cTBS, continuous theta burst stimulation; DSM, Diagnostic and Statistical Manual of Mental Disorders; dTMS, deep transcranial magnetic stimulation; GAD‐7, BAI: Beck Anxiety Inventory; HAMA, Hamilton anxiety scale; HAMD, Hamilton depression scale; HrTMS, high‐frequency repetitive transcranial magnetic stimulation; ICD, the International Classification of Diseases; iTBS, intermittent theta burst stimulation; MASQ, Mood and Anxiety Symptom Questionnaire; Mini, the Mini International Neuropsychiatric Interview; MMSE, mini mental status examination; MoCA, Montreal Cognitive Assessment; NR, not reported; OCDS, the Obsessive‐Compulsive Drinking Scale; PACS, the Penn Alcohol Craving Scale; rTMS, repetitive transcranial magnetic stimulation; STAI, State‐Trait Anxiety Inventory Scale; TMS, transcranial magnetic stimulation; VAS, Visual Analog Scale; Zung, Zung Self‐Rating Depression Scale.

**TABLE 2:** Details for TMS.

First author	Publication year	Location	Intensity (% of MT)	Frequency (Hz)	Total pluses	Sessions	Time	Duration
Mishra	2010	rDLPFC	110	10	1000	10	4.9 s per train, inter‐train interval of 30 s	4W
Herremans	2012	rDLPFC	110	20	1560	1	1.9 s duration, inter‐train interval of 12 s	1D
Herremans	2013	rDLPFC	110	20	1560	1	1.9 s duration, inter‐train interval of 12 s	1D
Herremans	2015	rDLPFC	110	20	1560	15	1.9 s duration, inter‐train interval of 12 s	4D
Ceccanti	2015	mPFC	120	20	NR	10	50 stimuli delivered, inter‐train interval of 30 s	2W
Addolorato	2017	DLPFC bilateral	100	10	NR	12	50 pulses per train, inter‐train interval of 15 s	4W
Jansen	2019	rDLPFC	110	10	NR	2	5 s trains	NR
Perini	2020	rDLPFC	120	10	1500	15	50 trains were delivered, inter‐train interval of 20 s	3W
Raikwar	2020	lDLPFC	120	10	800	10	4 s per train, inter‐train interval of 26 s	10D
Feng	2022	lDLPFC	110	10	1530	14	inter‐train interval of 20 s	2W
Harel	2022	mPFC	100	10	3000	15	3 s per train, inter‐train interval of 15 s	3W
Hu	2022	G1: lDLPFC G2: rDLPFC	110	10	NR	10	5 s per train, inter‐train interval of 20 s	2W
Belgers	2022	rDLPFC	110	10	3000	10	5 s per train	10D
McCalley	2023	mPFC	30%–110%	50	3600	10	3 pulse burst, 5 Hz [200 ms] interburst intervals	10D
Hoven	2023	rDLPFC	110	10	NR	10	5 s per train	10D
Padula	2024	lDLPFC	85%–100%–110%	50	1200	20	5 pulses/s, 20 ms interpulse interval; 30 pulses/train, 8‐s intertrain interval, 20 trains	3W
Durazzo	2025	lDLPFC	110	50	1200	20	5 pulses/s, 20 ms interpulse interval	2W
Wang	2025	lDLPFC	NR	50	600	10	5 Hz repetition (2 s stimulation, 8 s interval)	5D
Wang	2025	lDLPFC	100%	10	NR	20	4 s per train, inter‐train interval of 26 s	4W

Abbreviations: D, day; lDLPFC, left dorsolateral prefrontal cortices; mPFC, medial prefrontal cortex; NR, not reported; rDLPFC, right dorsolateral prefrontal cortices; S, second; W, week.

### 3.3 Risk of Bias Assessment of Included Studies

**FIGURE 2:**
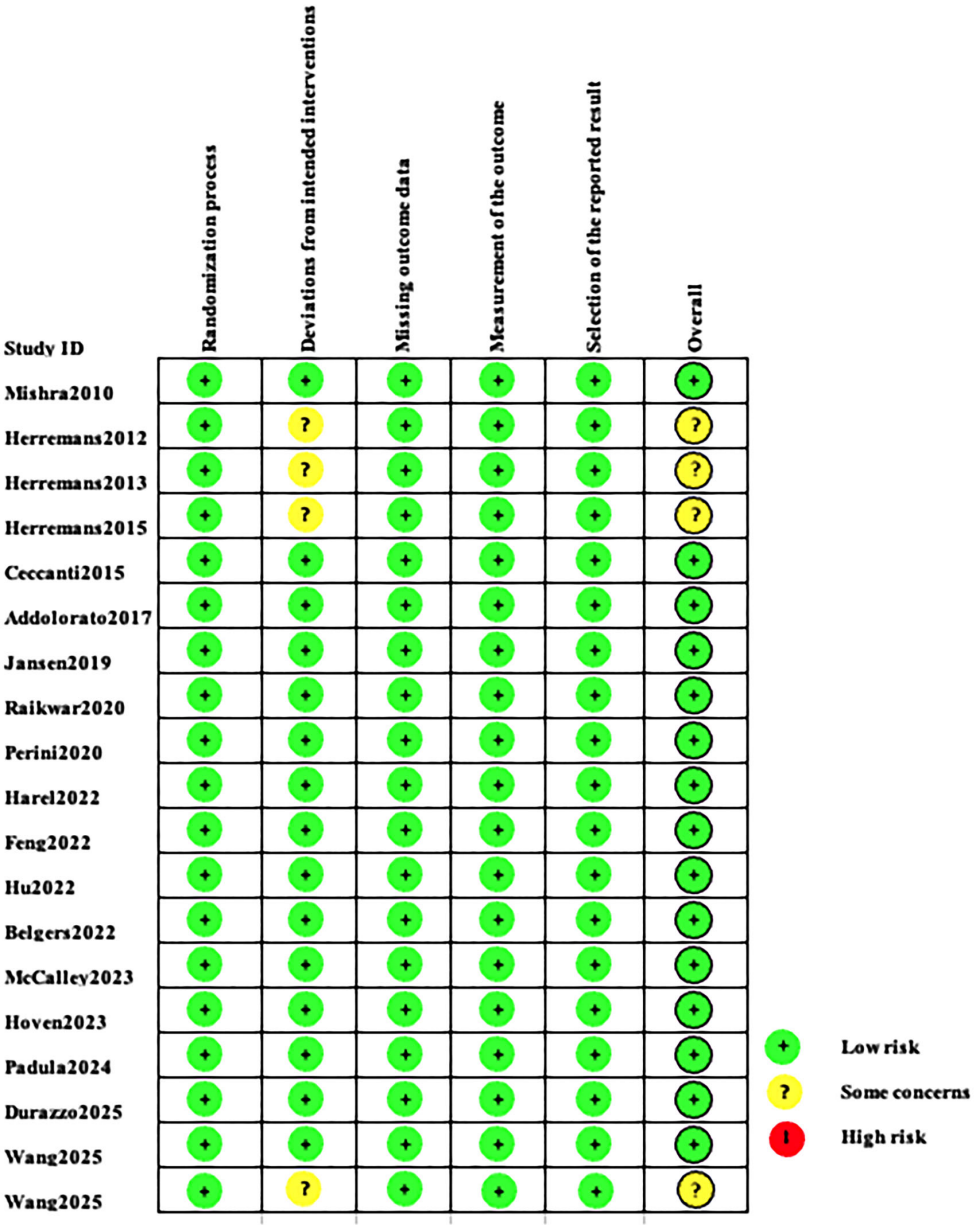
Risk of bias graph.

**FIGURE 3:**
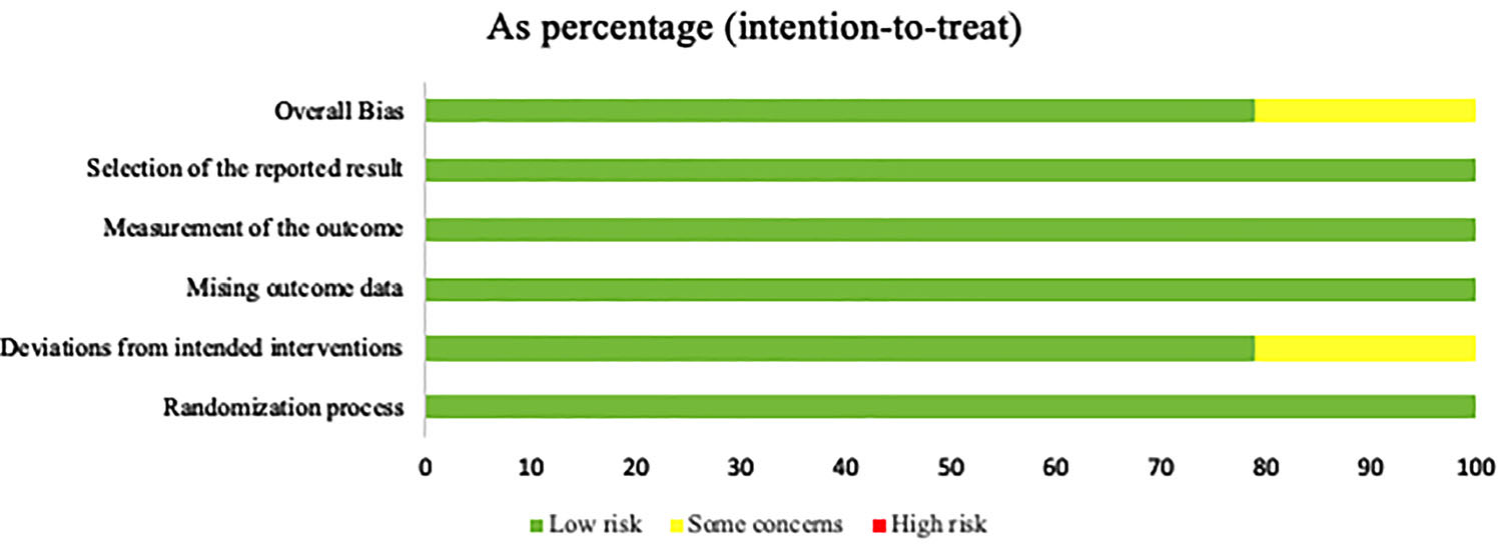
Summary of the risk of bias.

Based on the ROB2 assessment, four out of the 19 studies were rated as having a moderate risk of bias, and the remaining 15 were rated as having a low risk of bias. All included studies reported the randomization process, and 16 studies reported the blinding method. The main source of moderate risk of bias included bias arising from deviations from the intended interventions. See Figures 2 and 3.

### 3.4 Meta‐Analysis

#### 3.4.1 Effects of TMS on Alcohol Craving (Primary Outcome)

**FIGURE 4:**
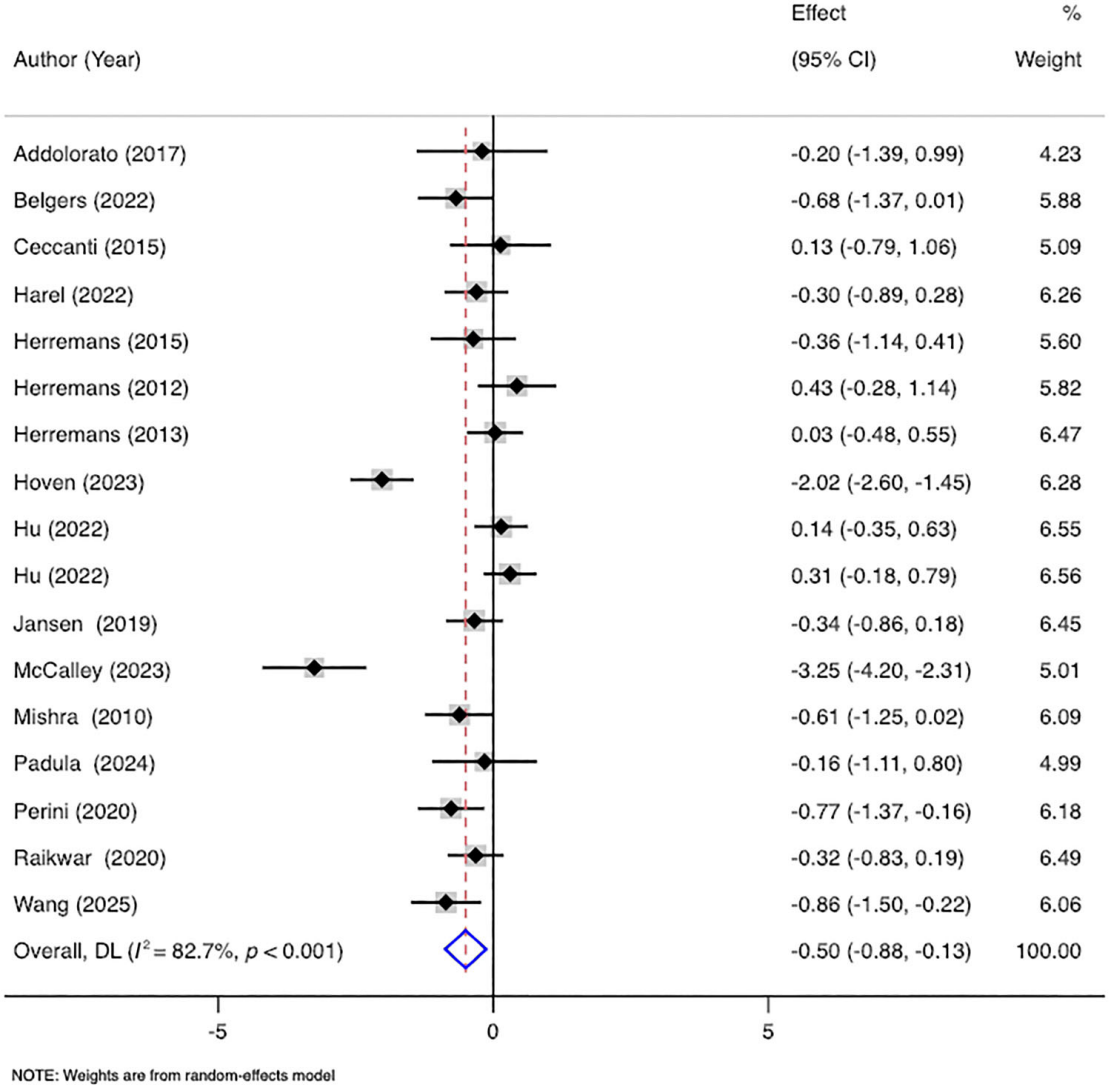
Forest plot of TMS intervention effect on alcohol craving. The red dashed line in the forest plot represents the pooled effect estimate obtained from the random‐effects model.

Sixteen studies evaluated the effect on alcohol craving in patients with AUD after their last TMS treatment. The results of the Cochrane *Q* test (*p* < 0.001) and *I* ^2^ (82.7%) estimation indicated significant heterogeneity. A meta‐analysis using a random‐effects model showed that TMS was more effective in reducing alcohol craving compared with sham stimulation (SMD = −0.50, 95% CI: −0.88, ‐0.13, *p* < 0.05). See Figure 4.

Further subgroup analyses were conducted based on characteristics such as stimulation parameters (site, mode, number of sessions, and frequency), follow‐up duration, age, country, assessment method, sample size, and disease type. The results showed that significant effect sizes were only observed in the following subgroups: TMS type as cTBS; number of sessions as 10 or 15; frequency as 10 Hz; follow‐up duration ≤ 1 month or ≥ 3 months; age < 45 years; country as India or Sweden; alcohol craving assessment tools as ACQ‐NOW or PACS; sample size ≥ 30; and diagnosis as AUD. See Table 3 and Figures S1–S10. Meta‐regression was performed on characteristics such as stimulation parameters (target, mode, number of sessions, frequency), age, country, sample size, and disease type (AUD or AD). The results showed that disease type (*p* = 0.04) might be the main reason for the high heterogeneity, while other factors showed no statistical significance. See Table 3 and Figures S11–S18.

**TABLE 3:** Subgroup analysis and meta‐regression results for TMS effects on alcohol craving.

Subgroup variable		*N*	Effect (95% CI)	*I* ^2^	Meta‐regression	References
**Stimulation site**					*p* = 0.96	
	DLPFC bilateral	1	−0.20 (−1.39, 0.99)	0%		Addolorato 2017
	rDLPFC	9	−0.47 (−0.94, 0.01)	82.4%		Belgers 2022; Herremans 2015; Herremans 2012; Herremans 2013; Hoven 2023; Hu 2022; Jansen 2019; Mishra 2010; Perini 2020
	mPFC	3	−1.13 (−3.00, 0.75)	93.8%		Ceccanti 2015; Harel 2022; McCalley 2023
	IDLPFC	4	−0.24 (−0.76, 0.28)	64.5%		Hu 2022; Padula 2024; Raikwar 2020; Wang 2025
**Stimulation mode**					*p* = 0.17	
	rTMS	6	−0.23 (−0.54, 0.08)	48.8%		Addolorato 2017; Belgers 2022; Hu 2022; Jansen 2019; Perini 2020; Raikwar 2020
	dTMS	2	−0.18 (−0.67, 0.31)	0%		Ceccanti 2015; Harel 2022
	hrTMS	5	−0.52 (−1.38, 0.35)	89.4%		Herremans 2015; Herremans 2012; Herremans 2013; Hoven 2023; Mishra 2010
	**cTBS**	1	**−3.25 (−4.20, −2.31)**	0%		McCalley 2023
	iTBS	2	−0.60 (−1.27, 0.06)	30.4%		Padula 2024; Wang 2025
**Stimulation sessions**					*p* = 0.87	
	< 10	3	−0.04 (−1.63, 1.55)	32.5%		Herremans 2012; Herremans 2013; Jansen 2019
	**10**	8	**−2.41 (−3.74, −1.08)**	95.8%		Belgers 2022; Ceccanti 2015; Hoven 2023; Hu 2022; McCalley 2023; Mishra 2010; Raikwar 2020; Wang 2025
	12	1	−2.70 (−19.12, 13.72)	0%		Addolorato 2017
	**15**	3	**−0.98 (−1.67, −0.29)**	0%		Harel 2022; Herremans 2015; Perini 2020
	20	1	−0.50 (−3.58, 2.58)	0%		Padula 2024
**Stimulation frequency**					*p* = 0.34	
	**10 Hz**	9	**−0.48 (−0.90, −0.06)**	80.5%		Addolorato 2017; Belgers 2022; Harel 2022; Hoven 2023; Hu 2022; Jansen 2019; Mishra 2010; Perini 2020; Raikwar 2020
	20 Hz	4	0.06 (−0.28, 0.40)	0%		Ceccanti 2015; Herremans 2015; Herremans 2012; Herremans 2013
	50 Hz	3	−1.41 (−3.08, 0.25)	91.5%		McCalley 2023; Padula 2024; Wang 2025
**Follow‐up duration**					/	
	**≤ 1 month**	16	**−0.50 (−0.88, −0.13)**	82.7%		Addolorato 2017; Belgers 2022; Ceccanti 2015; Harel 2022; Herremans 2015; Herremans 2012; Herremans 2013; Hoven 2023; Hu 2022; Jansen 2019; McCalley 2023; Mishra 2010; Padula 2024; Perini 2020; Raikwar 2020; Wang 2025
	1–3 months	6	−0.34 (−0.96, 0.29)†	84.8%		Belgers 2022; Ceccanti 2015; Hu 2022; McCalley 2023; Perini 2020; Harel 2022
	**≥ 3 months**	6	**−1.16 (−2.07, −0.26)**	93%		Belgers 2022; Hoven 2023; Hu 2022; McCalley 2023; Perini 2020; Harel 2022
**Age**					*p* = 0.82	
	≥ 45 years	6	−0.55 (−1.15, 0.05)	88.8%		Harel 2022; Jansen 2019; Mishra 2010; Padula 2024; Raikwar 2020; Wang 2025
	**< 45 years**	10	**−0.43 (−0.68, −0.19)**	0%		Addolorato 2017; Belgers 2022; Ceccanti 2015; Herremans 2015; Herremans 2012; Herremans 2013; Hoven 2023; Hu 2022; McCalley 2023; Perini 2020
**Country**					*p* = 0.73	
	Italy	2	0.01 (−0.72, 0.74)	0%		Addolorato 2017; Ceccanti 2015
	Netherlands	3	−1.02 (−2.07, 0.04)	89.5%		Belgers 2022; Hoven 2023; Jansen 2019
	Israel	1	−0.30 (−0.89, 0.28)	0%		Harel 2022
	Belgium	3	0.05 (−0.34, 0.44)	8.6%		Herremans 2015; Herremans 2012; Herremans 2013
	China	2	−0.11 (−0.75, 0.54)	77.1%		Hu 2022; Wang 2025
	America	2	−1.70 (−4.74, 1.33)	95.1%		McCalley 2023; Padula 2024
	**Sweden**	1	**−0.77 (−1.37, −0.16)**	0%		Perini 2020
	**India**	2	**−0.44 (−0.83, −0.04)**	0%		Mishra 2010; Raikwar 2020
**Assessment method**					/	
	AUQ	6	−0.95 (−1.99, 0.10)	93.5%		Perini 2020; McCalley 2023; Herremans 2015; Hoven 2023; Belgers 2022; Jansen 2019
	OCDS	8	−0.22 (−0.71, 0.26)	80.5%		Addolorato 2017; McCalley 2023; Hu 2022; Hoven 2023; Herremans 2012; Padula 2024; Herremans 2013; Herremans 2015
	**ACQ‐NOW**	2	**−0.44 (−0.83, −0.04)**	0%		Raikwar 2020; Mishra 2010
	**PACS**	2	**−0.53 (−0.98, −0.08)**	13.3%		Perini 2020; Harel 2022
	VAS	3	−0.84 (−1.79, 0.11)	77.4%		Wang 2025; Belgers 2022; Ceccanti 2015
**Sample size**					*p* = 0.27	
	< 30	4	−0.00 (−0.39, 0.38)	0%		Addolorato 2017; Ceccanti 2015; Herremans 2013; Padula 2024
	**≥ 30**	12	**−0.63 (−1.08, −0.18)**	86.2%		Belgers 2022; Harel 2022; Herremans 2015; Herremans 2012; Hoven 2023; Hu 2022; Jansen 2019; McCalley 2023; Mishra 2010; Perini 2020; Raikwar 2020; Wang 2025
**Disease type**					*p* = 0.04	
	**AUD**	6	**−1.12 (−2.04, −0.20)**	89%		Addolorato 2017; Belgers 2022; Hoven 2023; Jansen 2019; McCalley 2023; Padula 2024
	AD	10	−0.19 (‐0.45, 0.06)	49.5%		Ceccanti 2015; Harel 2022; Herremans 2015; Herremans 2012; Herremans 2013; Hu 2022; Mishra 2010; Perini 2020; Raikwar 2020; Wang 2025

Subgroups with statistically significant effect sizes (95% CI does not cross zero; P < 0.05) are shown in bold.

#### 3.4.2 Effects of TMS on Secondary Alcohol‐Related Outcomes and Comorbid Conditions

**FIGURE 5:**
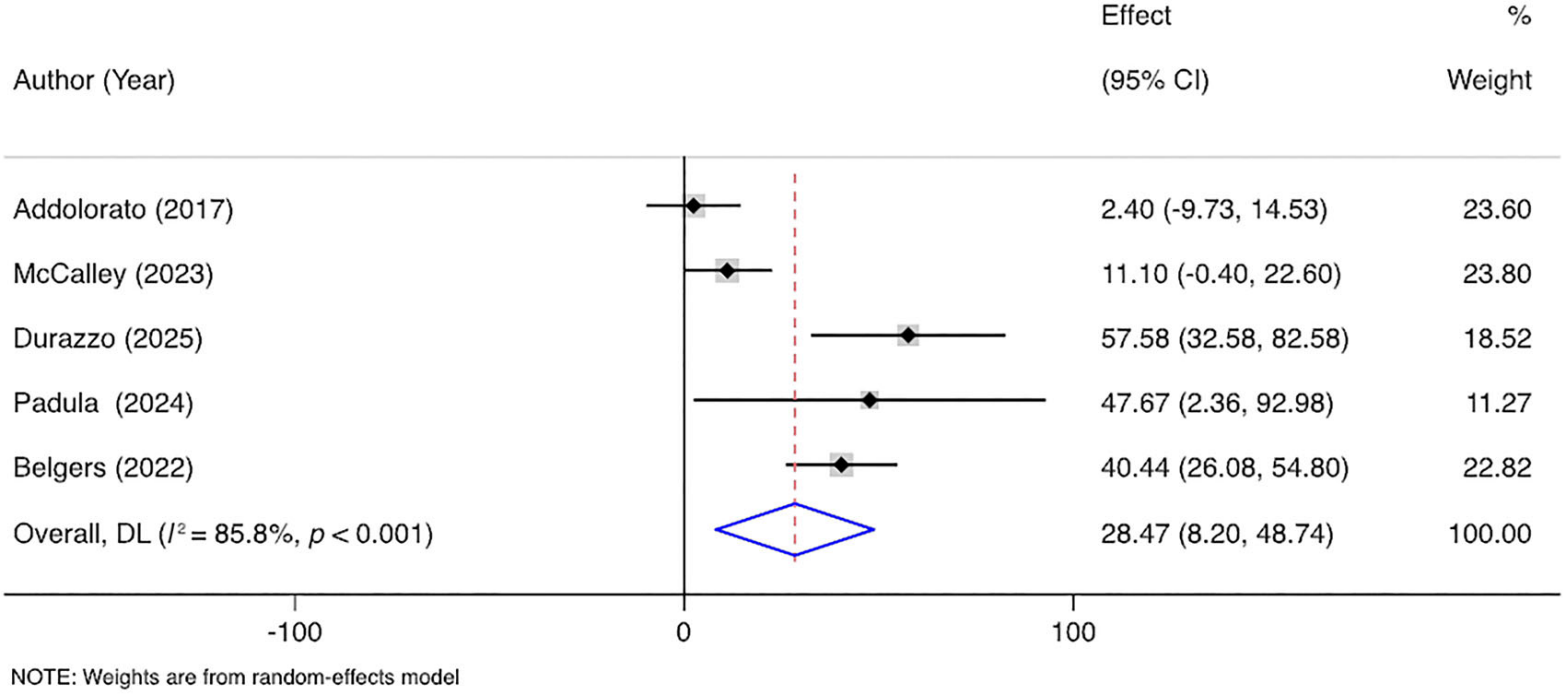
Forest plot of TMS intervention effect on abstinence days. The red dashed line in the forest plot represents the pooled effect estimate obtained from the random‐effects model.

**FIGURE 6:**
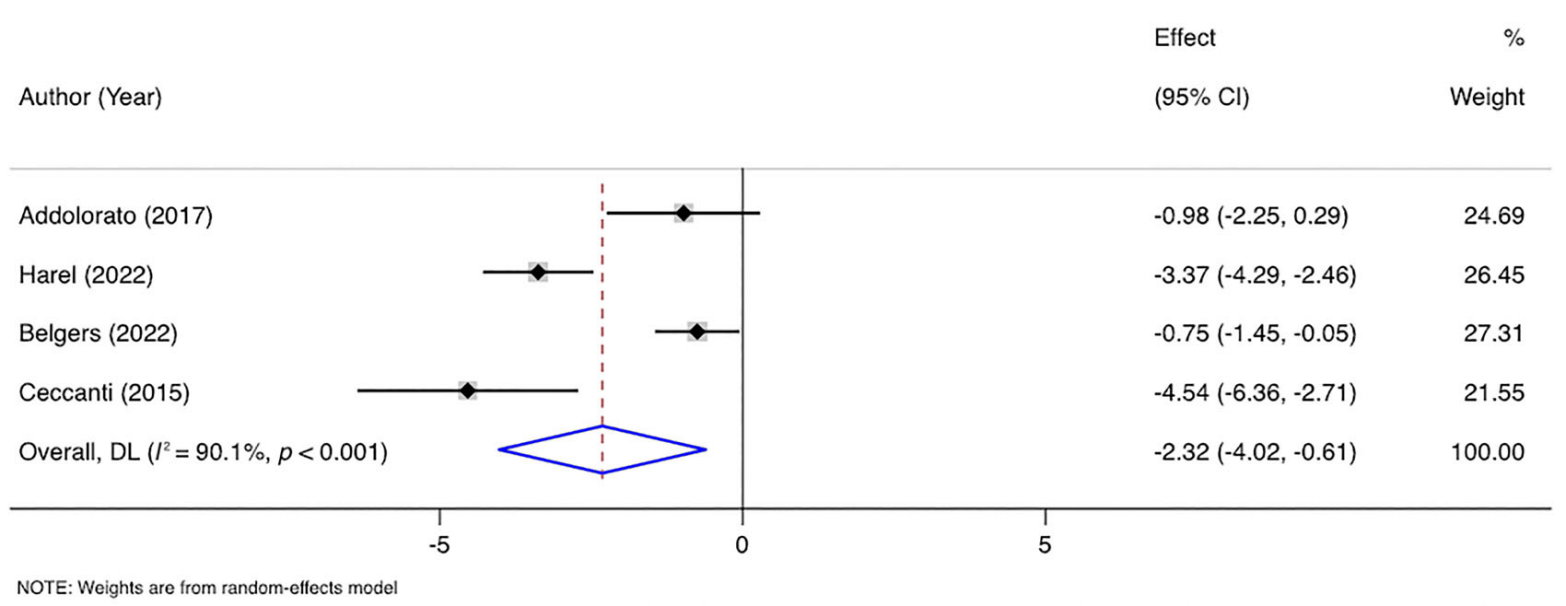
Forest plot of TMS intervention effect on alcohol intake. The red dashed line in the forest plot represents the pooled effect estimate obtained from the random‐effects model.

**FIGURE 7:**
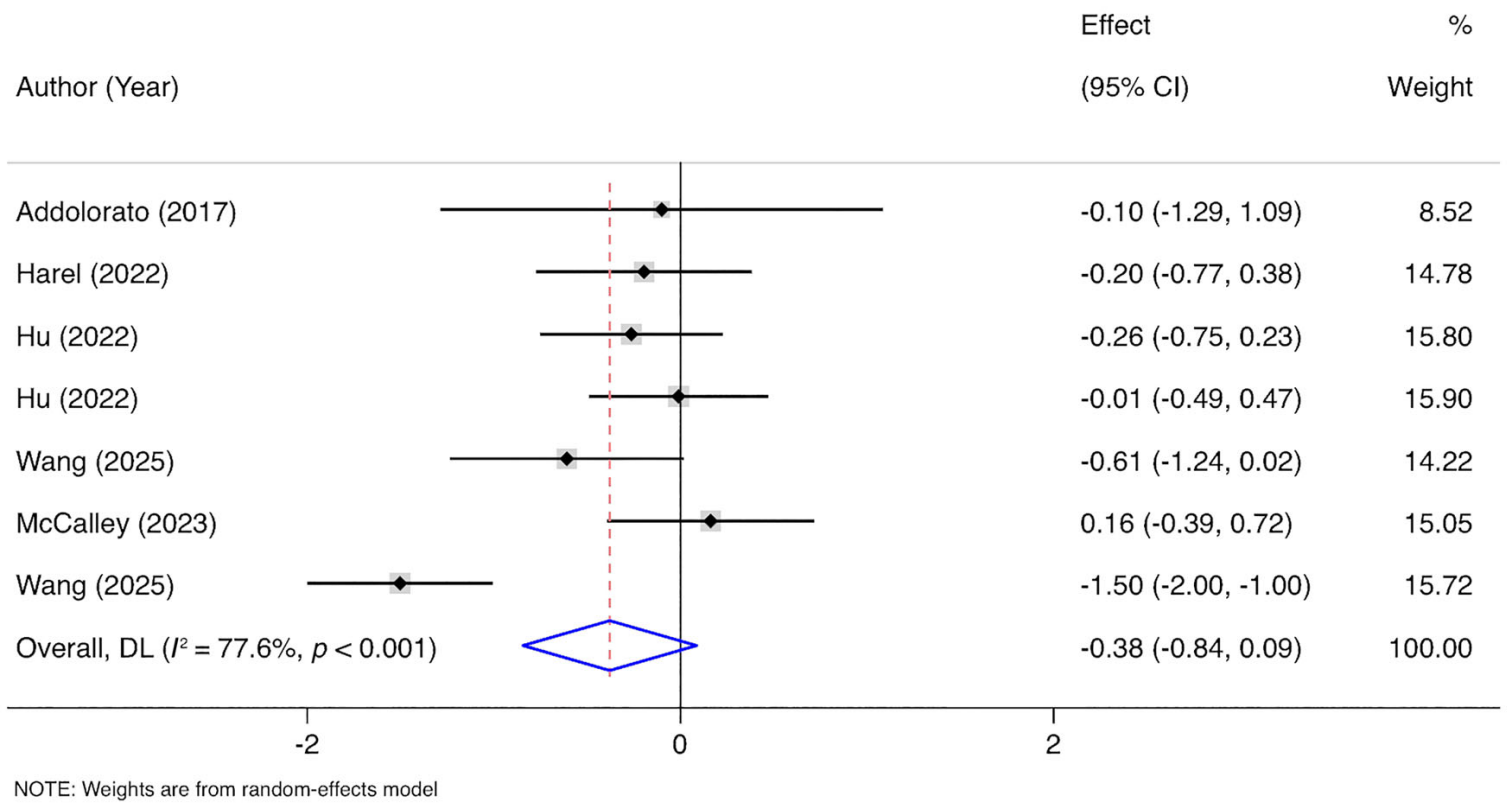
Forest plot of TMS intervention effect on anxiety. The red dashed line in the forest plot represents the pooled effect estimate obtained from the random‐effects model.

**FIGURE 8:**
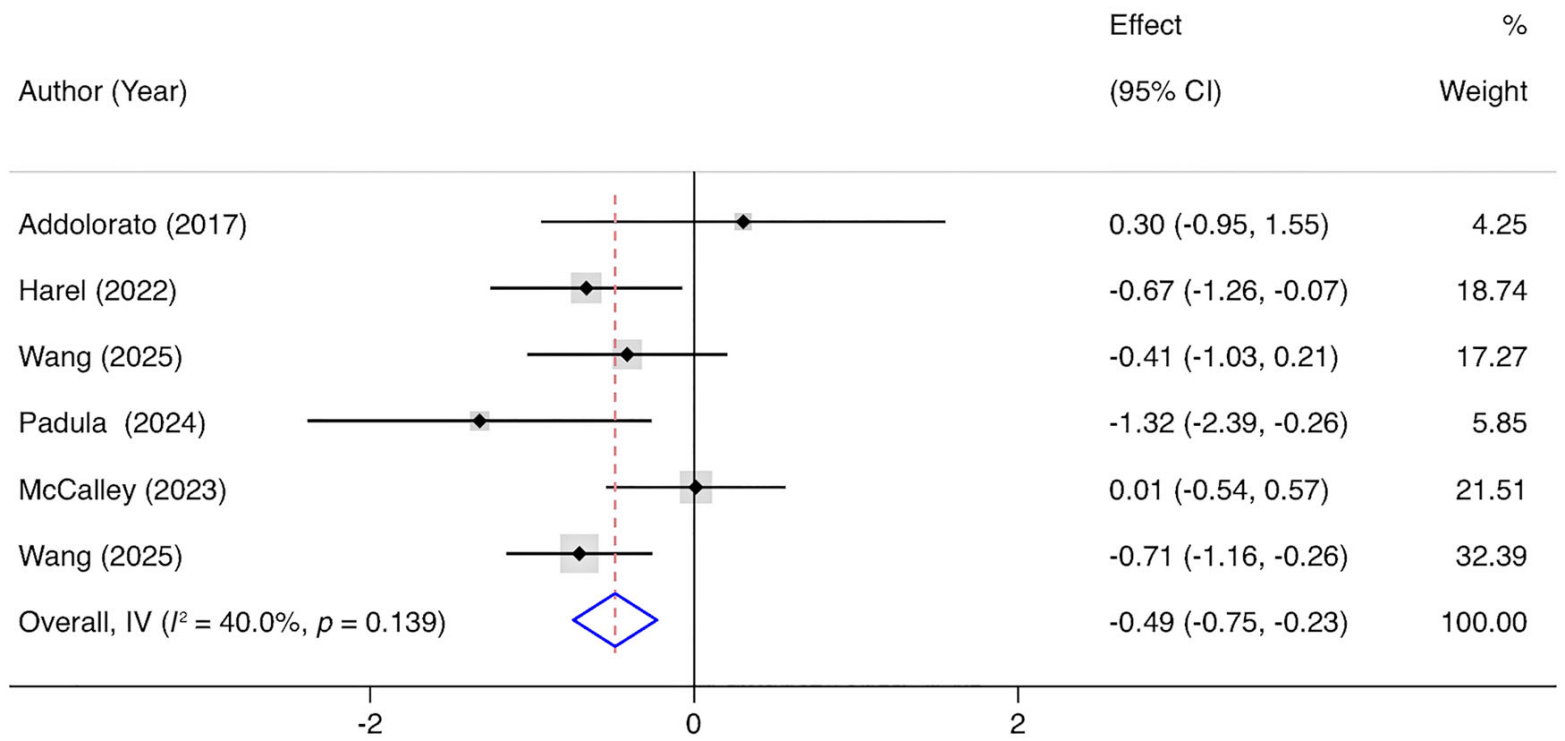
Forest plot of TMS intervention effect on depression. The red dashed line in the forest plot represents the pooled effect estimate obtained from the random‐effects model.

**FIGURE 9:**
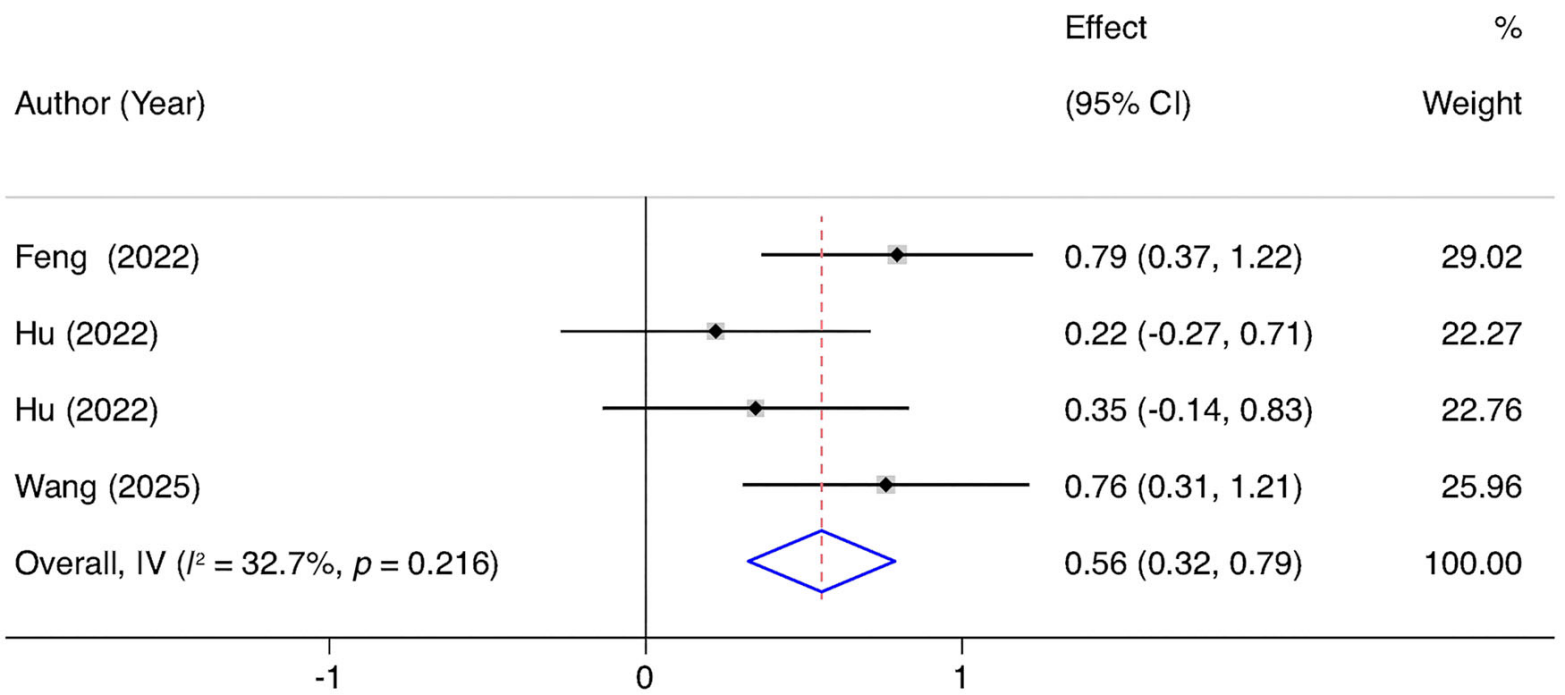
Forest plot of TMS intervention effect on cognitive function. The red dashed line in the forest plot represents the pooled effect estimate obtained from the random‐effects model.

To further evaluate the therapeutic efficacy of TMS, we analyzed outcomes across several key domains: alcohol abstinence days, alcohol consumption, anxiety, depression, and cognitive function. The results, summarized in Table 4 and Figures 5, 6, 7, 8, 9, indicate that TMS treatment demonstrated significant benefits in prolonging abstinence days and reducing alcohol intake compared to sham stimulation. Additionally, TMS was found to be more effective in alleviating depressive symptoms and improving cognitive function. However, no statistically significant difference was observed between TMS and sham groups in the reduction of anxiety levels.

**TABLE 4:** Overview of meta‐analytic findings for secondary outcomes (abstinence, alcohol consumption, and comorbid conditions) in patients with alcohol use disorder.

Variable	N	Cochrane *Q* test *p*‐value	*I* ^2^	Pooled effect size	95% CI	*p*‐value for effect	Effect model	Heterogeneity assessment
**Abstinence days**	5	*p* < 0.001	85.8%	SMD = 28.47	8.20, 48.74	*p* < 0.05	Random‐effects model	Significant heterogeneity
**Alcohol intake**	5	*p* < 0.001	90.1%	SMD = −2.32	−4.02, −0.61	*p* < 0.05	Random‐effects model	Significant heterogeneity
**Anxiety**	6	*p* < 0.001	77.6%	SMD = −0.38	−0.84, 0.09	*p* = 0.11	Random‐effects model	Significant heterogeneity
**Depression**	6	*p* = 0.14	40.0%	SMD = −0.49	−0.75, −0.23	*p* < 0.05	Fixed‐effects model	No significant heterogeneity
**Cognitive function**	3	*p* = 0.22	32.7%	MD = 0.56	0.32, 0.79	*p* < 0.05	Fixed‐effects model	No significant heterogeneity

### 3.5 Publication Bias

For the primary outcome of alcohol craving, publication bias was evaluated using a funnel plot and Egger's test. The funnel plot showed slight asymmetry, suggesting a potential risk of publication bias. Further Egger's test results showed a *p*‐value of 0.281, indicating no significant publication bias. See Figures S19 and S20.

### 3.6 Sensitivity Analysis

Sensitivity analyses were conducted for each outcome, and the results showed that excluding any single study had no substantial impact on changing the main conclusions. See Figures S21–S26.

## 4 Discussion

### 4.1 Principal Findings

To our knowledge, this is the first study to comprehensively evaluate the effects of TMS on alcohol craving, alcohol consumption, abstinence outcomes, mood, and cognition in patients with AUD through systematic review and meta‐analysis. This meta‐analysis, which included 19 RCTs, indicates that, compared with sham stimulation, TMS can significantly reduce alcohol craving in patients with AUD following intervention. Subgroup analyses suggest that the efficacy of TMS may vary according to stimulation parameters, patient characteristics, and follow‐up duration. Meta‐regression further indicates that disease type may be a major contributor to the observed heterogeneity. For secondary outcomes, TMS demonstrated significant benefits in prolonging abstinence days, reducing alcohol intake, and improving depressive symptoms and cognitive function, whereas no significant effect was observed for anxiety.

Subgroup analyses suggest that the efficacy of TMS may be influenced by stimulation parameters and patient characteristics. In particular, stimulation mode, session number, and stimulation frequency appear to play important roles in modulating treatment outcomes. These findings are consistent with the notion that optimized stimulation protocols may enhance neuroplasticity and improve treatment response. However, these results should be interpreted with caution given the limited number of studies in certain subgroups.

The effects of TMS also appeared to vary across follow‐up durations. Specifically, significant effects were observed at ≤ 1 month and ≥ 3 months, whereas no statistically significant effects were identified at intermediate follow‐up periods (e.g., 1–3 months). This pattern suggests that TMS may exert both short‐term and sustained therapeutic effects, while the absence of significant findings at intermediate time points may reflect between‐study heterogeneity or limited statistical power rather than a true lack of efficacy. Previous studies have reported similar trends. For example, Treiber et al. (2025) found that repetitive TMS produced only a moderate improvement in alcohol craving during a 3‐month follow‐up period. This finding supports the possibility that treatment effects may fluctuate over time and that evidence within intermediate follow‐up windows remains inconclusive due to the limited number of available studies.

The therapeutic effects of TMS on alcohol craving may be explained by its modulation of neurobiological mechanisms underlying addiction. AUD is a chronic relapsing disease. Even though patients receive abstinence treatment, there is still a possibility of relapse. The main factor leading to relapse is patients' craving for alcohol (Heinz et al. 2003). Alcohol craving and addiction may be related to brain network dysfunction. Tolomeo and Yu (2022) found that patients with substance use disorders show hyperconnectivity in the basal ganglia, insula, amygdala, and parahippocampal gyrus, indicating enhanced connectivity of the reward and salience networks. TMS affects changes in brain function (including reward processing, craving, and cognitive control) caused by excessive alcohol stimulation through magnetic pulses, inducing beneficial neuroplasticity (Moretti et al. 2020; Rossini and Rossi 2007). These neurobiological effects may ultimately contribute to reduced alcohol craving and improved self‐regulation in patients with AUD.

Subgroup analysis of alcohol craving based on TMS parameters showed that different stimulation parameters produced significant intervention effects. Among them, cTBS, an inhibitory protocol applied to the (mPFC), yielded the largest effect size (SMD = −3.25), whereas 10 Hz repetitive transcranial magnetic stimulation (10 Hz rTMS), an excitatory protocol mostly targeting the dorsolateral prefrontal cortex (DLPFC), also demonstrated significant efficacy but with a relatively smaller effect size (SMD = −2.41). cTBS is an innovative and unique variant of TMS (Zong et al. 2022). It can induce changes in neuroplasticity in a short time by applying fewer pulses and lower intensity (Chu et al. 2021). In addition, cTBS can reduce the brain's reactivity to alcohol and drug cues within the frontostriatal and frontoinsular circuits, thereby achieving the goal of reducing alcohol craving (Kearney‐Ramos et al. 2018). It is worth noting that only one eligible study adopted the cTBS paradigm in the included literature, so this optimal parameter conclusion should be interpreted with considerable caution due to the limited sample of relevant studies.

Regarding the number of sessions, single‐session, 2‐session, and 20‐session protocols did not show significant reductions in craving, whereas 10‐ and 15‐session protocols demonstrated significant effects, with the strongest effect observed at 10 sessions. Although a cumulative dose effect has been proposed, the lack of significant findings in the 20‐session subgroup suggests that the relationship between session number and treatment efficacy may not be strictly linear. This discrepancy may be attributed to several factors, including the limited number of studies in the 20‐session subgroup, differences in stimulation protocols, or variability in patient characteristics across studies. Therefore, the apparent “optimal” effect at 10 sessions should be interpreted cautiously rather than as evidence of a simple dose–response relationship. This may be related to the potential cumulative effects of repeated stimulation, which have been associated with long‐term potentiation (LTP) in previous studies (Herremans et al. 2012; Song et al. 2019). However, given the lack of significant findings in the 20‐session subgroup in our analysis, this effect may not follow a simple linear dose–response relationship and should be interpreted with caution. Regarding frequency, all studies adopted high‐frequency stimulation (at least 10 Hz), and significant intervention effects were only observed when the frequency was 10 Hz. Targeted TMS frequencies can participate in or induce local oscillatory activities of the brain to achieve the purpose of regulating brain rhythms (Q. Wang et al. 2024). Patients with different diseases have different oscillation mechanisms. For TMS treatment in patients with AUD, 10 Hz may represent a potentially effective stimulation frequency based on the current evidence; however, further studies are needed to confirm its optimality. In terms of stimulation targets, we did not find significant results for any stimulation target. Although most studies have shown that the DLPFC in the striatum and its related circuits are key neural circuits involved in executive control and the transition from drinking to AD (George and Koob 2010; Gremel and Costa 2013). This may be related to the severity of alcohol craving in patients with AUD and differences in other stimulation parameters.

Our study found that TMS can maintain alcohol abstinence days and reduce alcohol intake, which is consistent with the research by Addolorato et al. (2017). Addolorato et al. (2017) found that TMS can induce neurobiological changes in the mesolimbic and mesocortical striatal systems, specifically a reduction in dopamine transporter (DAT) availability. This reduces dopamine reuptake, decreases the sensitivity of the reward system to alcohol, and ultimately leads to a reduction in drinking behavior. In addition, TMS reduces alcohol intake by regulating the dopamine system (such as modifying prolactin and cortisol), thereby diminishing alcohol‐related reward effects (Ceccanti et al. 2015).

Our study found that TMS has a significant therapeutic effect on reducing depressive symptoms in patients with AUD. The improvement of depressive symptoms may, on the one hand, be related to the reduction of craving; on the other hand, it may alleviate depressive symptoms by applying TMS magnetic fields to specific regions related to emotional regulation and changing neuronal activity (Batista et al. 2015; Ebrahimzadeh et al. 2023; Feffer et al. 2022). In addition, we did not observe a significant effect of TMS on anxiety symptoms. This finding should be interpreted with caution, as concomitant pharmacological treatments may have influenced the results. Based on our review of the included studies, several trials explicitly reported the use of diazepam during alcohol withdrawal management, while others reported benzodiazepine use without specifying the compound, and a substantial proportion of studies did not provide detailed information on such medications. Given that benzodiazepines, including diazepam, have well‐established sedative and anxiolytic effects, their use may have contributed to the attenuation of differences in anxiety outcomes between the TMS and sham groups. However, due to inconsistent reporting across studies, this potential confounding effect could not be formally examined and should be interpreted cautiously.

Our study found that TMS has a significant therapeutic effect on improving cognitive function in patients with AUD, which is consistent with the findings of Feng et al. (2022). This study revealed that TMS reduced impulsivity and improved cognitive function, particularly in attention, memory, and orientation. This may be related to the fact that TMS induces neuroplasticity and accelerates the recovery of neural circuits (Cramer et al. 2011), and neuroplasticity is the most important mechanism in cognitive recovery (Crews et al. 2005). Ghin et al. (2022) suggested that using TMS as a potential complementary or alternative approach to regulate the activation of cortical and subcortical regions can help patients with AUD re‐establish a functional balance between controlled and automatic behaviors.

### 4.2 Limitations and Future Directions

Our study still has some limitations, and the results should be interpreted with caution. First, although we conducted subgroup analyses based on different stimulation parameters, factors such as gender, occupation, disease severity, AUD subtypes, and differences in the medications they received were still included in the analysis. Therefore, attention should be paid to clinical heterogeneity. Second, there are few studies investigating the long‐term maintenance effect of TMS on patients with AUD. It is recommended that future studies focus on the therapeutic effect over 6 months. Third, the sample size included in most studies is insufficient, or the number of studies included for a certain TMS type is too small, which may affect the credibility of the results. Fourth, most studies did not report the safety of TMS, which prevented us from collecting sufficient literature to verify its safety. Therefore, we look forward to including more high‐quality studies in the future. Based on big data methods such as machine learning, we aim to verify the optimal TMS neural targets, doses, and parameters; evaluate its safety; and further provide a reliable basis for clinical standardized protocols.

## 5 Conclusion

In conclusion, our meta‐analysis shows that TMS can effectively alleviate cravings in patients with AUD both in the short term and long term. When the TMS stimulation frequency is 10 times or 10 Hz, the intervention effect is optimal. TMS also demonstrates significant advantages in maintaining abstinence days, reducing alcohol intake, relieving depressive symptoms, and improving cognitive function. However, due to the limitations of this study, more large‐sample, multi‐center, double‐blind RCTs are needed in the future to supplement and validate the results of this study.

## Author Contributions

Conceptualization: **Jiayuan Song**. Methodology: **Ji Ouyang**. Software: Ji Ouyang. Validation: **Xingchen He**. Formal Analysis: Xingchen He. Investigation: Ji Ouyang. Resources: Jiayuan Song. Data Curation: Jiayuan Song. Writing – Original Draft Preparation: Ji Ouyang. Writing – Review and Editing: Jiayuan Song. Visualization: **Yanlin Liu**. Supervision: **Han Yu**. Project Administration: **Aimin Li**. Funding Acquisition: **Hongwei Liu** and **Haixia Fan**. Writing – Review and Editing: **Zhaoying Li**. Data Curation: Zhaoying Li. Writing – Review and Editing: **Fan Yao**. Supervision: Fan Yao. Project Administration: Fan Yao. Zhaoying Li renewed the database search, included newly published eligible studies, rechecked and verified all data extraction and analysis, and completed parts of the revised manuscript writing. Fan Yao made indispensable and substantial contributions to the revision of this manuscript, redesigned the revised framework, comprehensively polished and revised the full text, supplemented key discussion and interpretation of results, fully addressed all reviewer comments, drafted the point‐by‐point response letter, and took full charge of all subsequent journal communication and follow‐up work. All authors have read and agreed to the published version of the manuscript.

## Funding

This work was supported by the Shanxi Health Committee Foundation Project (2024057), the Fundamental Research Program of Shanxi Province (202403021222432), and the Jilin Provincial Natural Science Foundation of China (Grant No. YDZJ202601ZYTS670).

## Ethics Statement

This study utilized publicly available summary data, and according to relevant guidelines, ethics approval was not required. The use of open‐access data meant that individual privacy was safeguarded, eliminating the need for additional ethical review.

## Consent

The authors have nothing to report.

## Supporting information




**Figure S1** Subgroup analysis based on stimulation site
**Figure S2** Subgroup analysis based on stimulation mode
**Figure S3** Subgroup analysis based on stimulation sessions
**Figure S4** Subgroup analysis based on stimulation frequency
**Figure S5** Subgroup analysis based on follow‐up duration
**Figure S6** Subgroup analysis based on age
**Figure S7** Subgroup analysis based on country
**Figure S8** Subgroup analysis based on assessment method
**Figure S9** Subgroup analysis based on f sample size
**Figure S10** Subgroup analysis based on disease type
**Figure S11** Meta regression based on stimulation site
**Figure S12** Meta regression based on stimulation technique
**Figure S13** Meta regression based on stimulation sessions
**Figure S14** Meta regression based on stimulation frequency
**Figure S15** Meta regression based on age
**Figure S16** Meta regression based on country
**Figure S17** Meta regression based on sample size
**Figure S18** Meta regression based on disease type
**Figure S19** The funnel plot of alcohol craving
**Figure S20** Egger's test for alcohol craving
**Figure S21** Sensitivity analysis of alcohol craving
**Figure S22** Sensitivity analysis of abstinence days
**Figure S23** Sensitivity analysis of alcohol intake
**Figure S24** Sensitivity analysis of anxiety
**Figure S25** Sensitivity analysis of depression
**Figure S26** Sensitivity analysis of cognitive function

## Data Availability

This systematic review and meta‐analysis used data extracted from previously published studies. All effect sizes, covariates, and study characteristics used in our analysis are reported in the main text or supplementary materials. We did not generate any new individual‐level dataset; therefore, no data repository link is provided. Raw participant‐level data are not available from us and should be requested from the original journals via the references cited. For inquiries regarding data extraction procedures or analytical codes, please contact the corresponding author, Fan Yao (13258841878@163.com). The datasets used and analyzed during the current study are available from the corresponding author upon reasonable request.
