# Supplementary material for: The Effects of Transcranial Magnetic Stimulation on Alcohol Craving, Efficacy, Emotion, and Cognitive Function in Patients With Alcohol Use Disorders: A Systematic Review and Meta‐Analysis of Randomized Controlled Trial
Source: Brain Behav. 2026 Jul 26;16(7):e71605. doi: 10.1002/brb3.71605 (PMC13402854; doi:10.1002/brb3.71605)
Supplement: Supplementary file 1 — Figure S1 Subgroup analysis based on stimulation site Figure S2 Subgroup analysis based on stimulation mode Figure S3 Subgroup analysis based on stimulation sessions Figure S4 Subgroup analysis based on stimulation frequency Figure S5 Subgroup analysis based on follow‐up duration Figure S6 Subgroup analysis based on age Figure S7 Subgroup analysis based on country Figure S8 Subgroup analysis based on assessment method Figure S9 Subgroup analysis based on f sample size Figure S10 Subgroup analysis based on disease type Figure S11 Meta regression based on stimulation site Figure S12 Meta regression based on stimulation technique Figure S13 Meta regression based on stimulation sessions Figure S14 Meta regression based on stimulation frequency Figure S15 Meta regression based on age Figure S16 Meta regression based on country Figure S17 Meta regression based on sample size Figure S18 Meta regression based on disease type Figure S19 The funnel plot of alcohol craving Figure S20 Egger's test for alcohol craving Figure S21 Sensitivity analysis of alcohol craving Figure S22 Sensitivity analysis of abstinence days Figure S23 Sensitivity analysis of alcohol intake Figure S24 Sensitivity analysis of anxiety Figure S25 Sensitivity analysis of depression Figure S26 Sensitivity analysis of cognitive function [file BRB3-16-e71605-s001.pdf]

**Table S1: PRISMA 2020 statement**

| Section and Topic | Item # | Checklist item | Location where item is reported |
|---|---|---|---|
| **TITLE** | | | |
| Title | 1 | Identify the report as a systematic review. | 1 |
| **ABSTRACT** | | | |
| Abstract | 2 | See the PRISMA 2020 for Abstracts checklist. | 1 |
| **INTRODUCTION** | | | |
| Rationale | 3 | Describe the rationale for the review in the context of existing knowledge. | 2-3 |
| Objectives | 4 | Provide an explicit statement of the objective(s) or question(s) the review addresses. | 4 |
| **METHODS** | | | |
| Eligibility criteria | 5 | Specify the inclusion and exclusion criteria for the review and how studies were grouped for the syntheses. | 4 |
| Information sources | 6 | Specify all databases, registers, websites, organisations, reference lists and other sources searched or consulted to identify studies. Specify the date when each source was last searched or consulted. | 4 |
| Search strategy | 7 | Present the full search strategies for all databases, registers and websites, including any filters and limits used. | 4 |
| Selection process | 8 | Specify the methods used to decide whether a study met the inclusion criteria of the review, including how many reviewers screened each record and each report retrieved, whether they worked independently, and if applicable, details of automation tools used in the process. | 5 |
| Data collection process | 9 | Specify the methods used to collect data from reports, including how many reviewers collected data from each report, whether they worked independently, any processes for obtaining or confirming data from study investigators, and if applicable, details of automation tools used in the process. | 5 |
| Data items | 10a | List and define all outcomes for which data were sought. Specify whether all results that were compatible with each outcome domain in each study were sought (e.g. for all measures, time points, analyses), and if not, the methods used to decide which results to collect. | 5-6 |
| | 10b | List and define all other variables for which data were sought (e.g. participant and intervention characteristics, funding sources). Describe any assumptions made about any missing or unclear information. | 6 |
| Study risk of bias assessment | 11 | Specify the methods used to assess risk of bias in the included studies, including details of the tool(s) used, how many reviewers assessed each study and whether they worked independently, and if applicable, details of automation tools used in the process. | 6 |
| Effect measures | 12 | Specify for each outcome the effect measure(s) (e.g. risk ratio, mean difference) used in the synthesis or presentation of results. | 6 |
| Synthesis methods | 13a | Describe the processes used to decide which studies were eligible for each synthesis (e.g. tabulating the study intervention characteristics and comparing against the planned groups for each synthesis (item #5)). | 6 |
| | 13b | Describe any methods required to prepare the data for presentation or synthesis, such as handling of missing summary statistics, or data conversions. | 6 |
| | 13c | Describe any methods used to tabulate or visually display results of individual studies and syntheses. | 6 |
| | 13d | Describe any methods used to synthesize results and provide a rationale for the choice(s). If meta-analysis was performed, describe the model(s), method(s) to identify the presence and extent of statistical heterogeneity, and software package(s) used. | 6 |
| | 13e | Describe any methods used to explore possible causes of heterogeneity among study results (e.g. subgroup analysis, meta-regression). | 6-7 |
| | 13f | Describe any sensitivity analyses conducted to assess robustness of the synthesized results. | 6-7 |
| Reporting bias assessment | 14 | Describe any methods used to assess risk of bias due to missing results in a synthesis (arising from reporting biases). | 7 |
| Certainty | 15 | Describe any methods used to assess certainty (or confidence) in the body of evidence for an outcome. | 7 |

| Section and Topic | Item # | Checklist item | Location where item is reported |
|---|---|---|---|
| assessment | | | |
| **RESULTS** | | | |
| Study selection | 16a | Describe the results of the search and selection process, from the number of records identified in the search to the number of studies included in the review, ideally using a flow diagram. | 7 |
| | 16b | Cite studies that might appear to meet the inclusion criteria, but which were excluded, and explain why they were excluded. | 7 |
| Study characteristics | 17 | Cite each included study and present its characteristics. | 7 |
| Risk of bias in studies | 18 | Present assessments of risk of bias for each included study. | 7 |
| Results of individual studies | 19 | For all outcomes, present, for each study: (a) summary statistics for each group (where appropriate) and (b) an effect estimate and its precision (e.g. confidence/credible interval), ideally using structured tables or plots. | 7 |
| Results of syntheses | 20a | For each synthesis, briefly summarise the characteristics and risk of bias among contributing studies. | 8 |
| | 20b | Present results of all statistical syntheses conducted. If meta-analysis was done, present for each the summary estimate and its precision (e.g. confidence/credible interval) and measures of statistical heterogeneity. If comparing groups, describe the direction of the effect. | 8 |
| | 20c | Present results of all investigations of possible causes of heterogeneity among study results. | 8 |
| | 20d | Present results of all sensitivity analyses conducted to assess the robustness of the synthesized results. | 8 |
| Reporting biases | 21 | Present assessments of risk of bias due to missing results (arising from reporting biases) for each synthesis assessed. | 9 |
| Certainty of evidence | 22 | Present assessments of certainty (or confidence) in the body of evidence for each outcome assessed. | 9 |
| **DISCUSSION** | | | |
| Discussion | 23a | Provide a general interpretation of the results in the context of other evidence. | 10 |
| | 23b | Discuss any limitations of the evidence included in the review. | 11 |
| | 23c | Discuss any limitations of the review processes used. | 11 |
| | 23d | Discuss implications of the results for practice, policy, and future research. | 11 |
| **OTHER INFORMATION** | | | |
| Registration and protocol | 24a | Provide registration information for the review, including register name and registration number, or state that the review was not registered. | 4 |
| | 24b | Indicate where the review protocol can be accessed, or state that a protocol was not prepared. | 4 |
| | 24c | Describe and explain any amendments to information provided at registration or in the protocol. | 4 |
| Support | 25 | Describe sources of financial or non-financial support for the review, and the role of the funders or sponsors in the review. | 11 |
| Competing interests | 26 | Declare any competing interests of review authors. | 12 |
| Availability of data, code and other materials | 27 | Report which of the following are publicly available and where they can be found: template data collection forms; data extracted from included studies; data used for all analyses; analytic code; any other materials used in the review. | 12 |

*From:* Page MJ, McKenzie JE, Bossuyt PM, Boutron I, Hoffmann TC, Mulrow CD, et al. The PRISMA 2020 statement: an updated guideline for reporting systematic reviews. BMJ 2021;372:n71. doi: 10.1136/bmj.n71

**Table S2: Search strategy**

**Cochrane**

ID Search Hits
#1 MeSH descriptor: [Blood Alcohol Content] explode all trees 87
#2 MeSH descriptor: [Alcoholic Intoxication] explode all trees 896
#3 MeSH descriptor: [Alcohols] explode all trees 45952
#4 MeSH descriptor: [Drinking Behavior] explode all trees 6157
#5 (alcoholism OR alcohol drink* OR alcohol intoxication OR alcohol abuse OR alcohol dependen* OR alcohol use OR alcohol addiction OR alcohol consum* OR binge drink* OR heavy drink*):ti,ab,kw OR (alcoholi* OR alcohol misuse OR alcohol abstinen* OR alcohol intake OR blood alcohol OR drunkenness* OR alcohol related OR hazardous drinking OR harmful drinking):ti,ab,kw 40162
#6 #1 OR #2 OR #3 OR #4 OR #5 82352
#7 MeSH descriptor: [Transcranial Magnetic Stimulation] explode all trees 3020
#8 (Transcranial Magnetic Stimulation* OR transcranial magnetic stimulat* OR noninvasive brain stimulation OR TMS OR TBS OR Theta Burst Stimulation):ti,ab,kw 14509
#9 #7 OR #8 14509
#10 (random* OR clinic* OR control OR trial OR placebo):ti,ab,kw 1957836
#11 #6 AND #9 AND #10 316

**Pubmed**

| Search number | Search Details | Results |
|---|---|---|
| 4:1 and 2 and 3 | ("random*"[Title/Abstract] OR "clinic*"[Title/Abstract] OR "control"[Title/Abstract] OR "trial"[Title/Abstract] OR "placebo"[Title/Abstract]) AND ("transcranial magnetic stimulation"[MeSH Terms] OR "transcranial magnetic stimulation*"[Title/Abstract] OR "transcranial magnetic stimulat*"[Title/Abstract] OR "noninvasive brain stimulation"[Title/Abstract] OR "theta burst stimulation"[Title/Abstract] OR "TMS"[Title/Abstract] OR "TBS"[Title/Abstract]) AND ("blood alcohol content"[MeSH Terms] OR "alcoholic intoxication"[MeSH Terms] OR "alcoholism"[MeSH Terms] OR "drinking behavior"[MeSH Terms] OR "alcoholism"[Title/Abstract] OR "alcohol drink*"[Title/Abstract] OR "alcohol intoxication"[Title/Abstract] OR "alcohol abuse"[Title/Abstract] OR "alcohol dependen*"[Title/Abstract] OR "alcohol use"[Title/Abstract] OR "alcohol addiction"[Title/Abstract] OR "alcohol consum*"[Title/Abstract] OR "binge drink*"[Title/Abstract] OR "heavy drink*"[Title/Abstract] OR "alcoholi*"[Title/Abstract] OR "alcohol misuse"[Title/Abstract] OR "alcohol abstinen*"[Title/Abstract] OR "alcohol intake"[Title/Abstract] OR "blood alcohol"[Title/Abstract] OR "drunkenness*"[Title/Abstract] OR "alcohol related"[Title/Abstract] OR "hazardous drinking"[Title/Abstract] OR "harmful drinking"[Title/Abstract]) | 149 |
| 3 | "random*"[Title/Abstract] OR "clinic*"[Title/Abstract] OR "control"[Title/Abstract] OR "trial"[Title/Abstract] OR "placebo"[Title/Abstract] | 10,191,691 |

| | | |
|---|---|---|
| 2 | "transcranial magnetic stimulation"[MeSH Terms] OR "transcranial magnetic stimulation*"[Title/Abstract] OR "transcranial magnetic stimulat*"[Title/Abstract] OR "noninvasive brain stimulation"[Title/Abstract] OR "theta burst stimulation"[Title/Abstract] OR "TMS"[Title/Abstract] OR "TBS"[Title/Abstract] | 39,268 |
| 1 | "blood alcohol content"[MeSH Terms] OR "alcoholic intoxication"[MeSH Terms] OR "alcoholism"[MeSH Terms] OR "drinking behavior"[MeSH Terms] OR "alcoholism"[Title/Abstract] OR "alcohol drink*"[Title/Abstract] OR "alcohol intoxication"[Title/Abstract] OR "alcohol abuse"[Title/Abstract] OR "alcohol dependen*"[Title/Abstract] OR "alcohol use"[Title/Abstract] OR "alcohol addiction"[Title/Abstract] OR "alcohol consum*"[Title/Abstract] OR "binge drink*"[Title/Abstract] OR "heavy drink*"[Title/Abstract] OR "alcoholi*"[Title/Abstract] OR "alcohol misuse"[Title/Abstract] OR "alcohol abstinen*"[Title/Abstract] OR "alcohol intake"[Title/Abstract] OR "blood alcohol"[Title/Abstract] OR "drunkenness*"[Title/Abstract] OR "alcohol related"[Title/Abstract] OR "hazardous drinking"[Title/Abstract] OR "harmful drinking"[Title/Abstract] | 320,721 |

**Embase**

| No. | Query | Results |
|---|---|---|
| #4 | #1 AND #2 AND #3 | 414 |
| #3 | random*:ab,ti OR clinic*:ab,ti OR control:ab,ti OR trial:ab,ti OR placebo:ab,ti | 14,493,154 |
| #2 | 'transcranial magnetic stimulation'/exp OR 'transcranial magnetic stimulation*':ab,ti OR 'transcranial magnetic stimulat*':ab,ti OR 'noninvasive brain stimulation':ab,ti OR tms:ab,ti OR tbs:ab,ti OR 'theta burst stimulation':ab,ti | 63,085 |
| #1 | 'blood alcohol content'/exp OR 'alcoholic intoxication'/exp OR 'alcoholism'/exp OR 'drinking behavior'/exp OR alcoholism:ab,ti OR 'alcohol drink*':ab,ti OR 'alcohol intoxication':ab,ti OR 'alcohol abuse':ab,ti OR 'alcohol dependen*':ab,ti OR 'alcohol use':ab,ti OR 'alcohol addiction':ab,ti OR 'alcohol consum*':ab,ti OR 'binge drink*':ab,ti OR 'heavy drink*':ab,ti OR alcoholi*:ab,ti OR 'alcohol misuse':ab,ti OR 'alcohol abstinen*':ab,ti OR 'alcohol intake':ab,ti OR 'blood alcohol':ab,ti OR drunkenness*:ab,ti OR 'alcohol related':ab,ti OR 'hazardous drinking':ab,ti OR 'harmful drinking':ab,ti | 465,565 |

**Web of Science**

1: alcoholism OR alcohol drink* OR alcohol intoxication OR alcohol abuse OR alcohol dependen* OR alcohol use OR alcohol addiction OR alcohol consum* OR binge drink* OR heavy drink* (Topic) OR (alcoholi* OR alcohol misuse OR alcohol abstinen* OR alcohol intake OR blood alcohol OR drunkenness* OR alcohol related OR hazardous drinking OR harmful drinking) (Topic) OR blood alcohol content OR alcoholic intoxication OR Alcohols OR drinking behavior (Topic) Results: 465020

2: Transcranial Magnetic Stimulation* OR transcranial magnetic stimulat* OR noninvasive brain stimulation OR TMS OR TBS OR Theta Burst Stimulation (Topic) Results: 47202

3: random* OR clinic* OR control OR trial OR placebo (Topic) Results: 10623359

4: #1 AND #2 AND #3 Results: 314

**China National Knowledge Infrastructure (CNKI)  Results:101**

（主题：酒精＋酗酒＋饮酒＋酒+过量饮酒＋嗜酒＋酒精使用障碍＋酒精依赖＋酒精滥用）AND（主题：经颅磁刺激+TMS + TBS）AND（篇关摘：随机＋临床＋试验＋对照 ＋安慰剂＋常规治疗＋常规护理（精确））

**China Science Journal Database (VIP)  Results:89**

题名或关键词=酒精＋酗酒 ＋ 饮酒 ＋ 酒＋过量饮酒＋嗜酒+酒精使用障碍＋酒精依赖＋酒精滥用 AND 题名或关键词=经颅磁刺激+TMS +TBS+磁刺激 AND 摘要=随机＋临床＋试验＋对照＋安慰剂＋常规治疗＋常规护理

**Wanfang Database  Results:336**

主题:(酒精 OR 酗酒 OR 饮酒 OR 酒 OR 过量饮酒 OR 嗜酒 OR 酒精使用障碍 OR 酒精依赖 OR 酒精滥用) and 主题:(经颅磁刺激 OR TMS OR TBS OR 磁刺激) and 摘要:(随机 OR 临床 OR 试验 OR 对照 OR 安慰剂 OR 常规治疗 OR 常规护理)

**China Biomedical Literature Service System (CBM)  Results:91**

( "酒精"[常用字段:智能] OR "酗酒"[常用字段:智能] OR "饮酒"[常用字段:智能] OR "酒"[常用字段:智能] OR "过量饮酒"[常用字段:智能] OR "嗜酒"[常用字段:智能] OR "酒精使用障碍"[常用字段:智能] OR "酒精依赖"[常用字段:智能] OR "酒精滥用"[常用字段:智能]) AND( "经颅磁刺激"[常用字段:智能] OR "TMS"[常用字段:智能] OR "TBS"[常用字段:智能] OR "磁刺激"[常用字段:智能]) AND( "随机"[常用字段:智能] OR "临床"[常用字段:智能] OR "试验"[常用字段:智能] OR "对照"[常用字段:智能] OR "安慰剂"[常用字段:智能] OR "常规治疗"[常用字段:智能] OR "常规护理"[常用字段:智能])

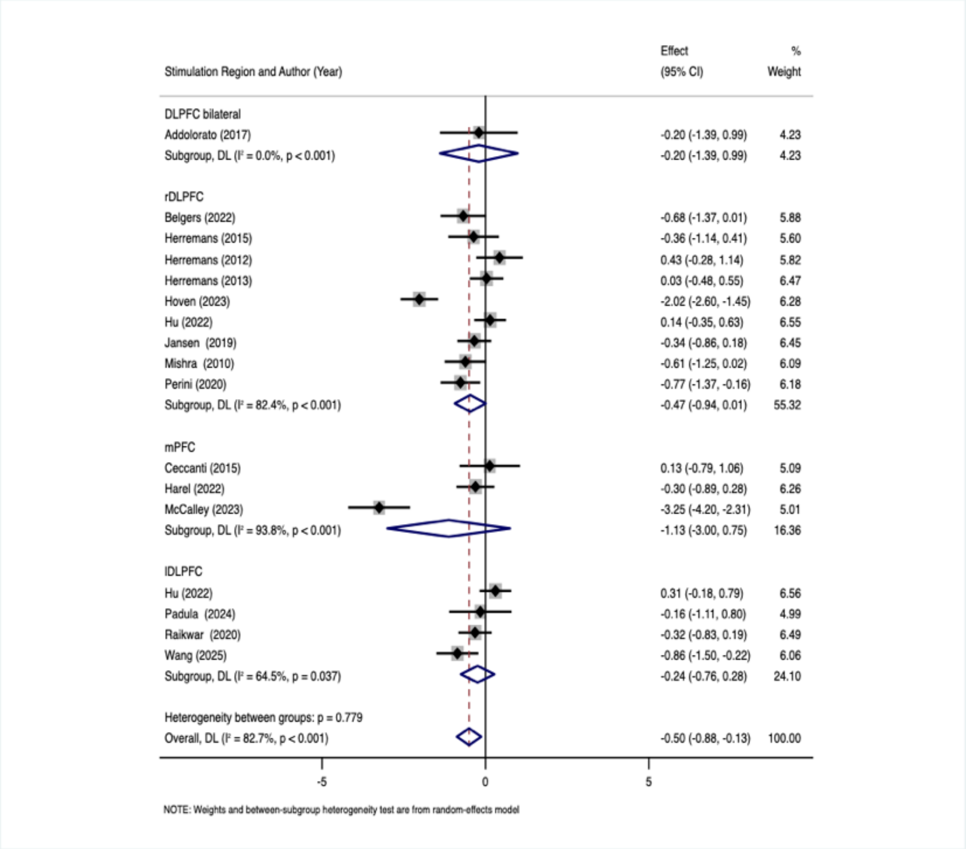


**Figure S1 Subgroup analysis based on stimulation site**

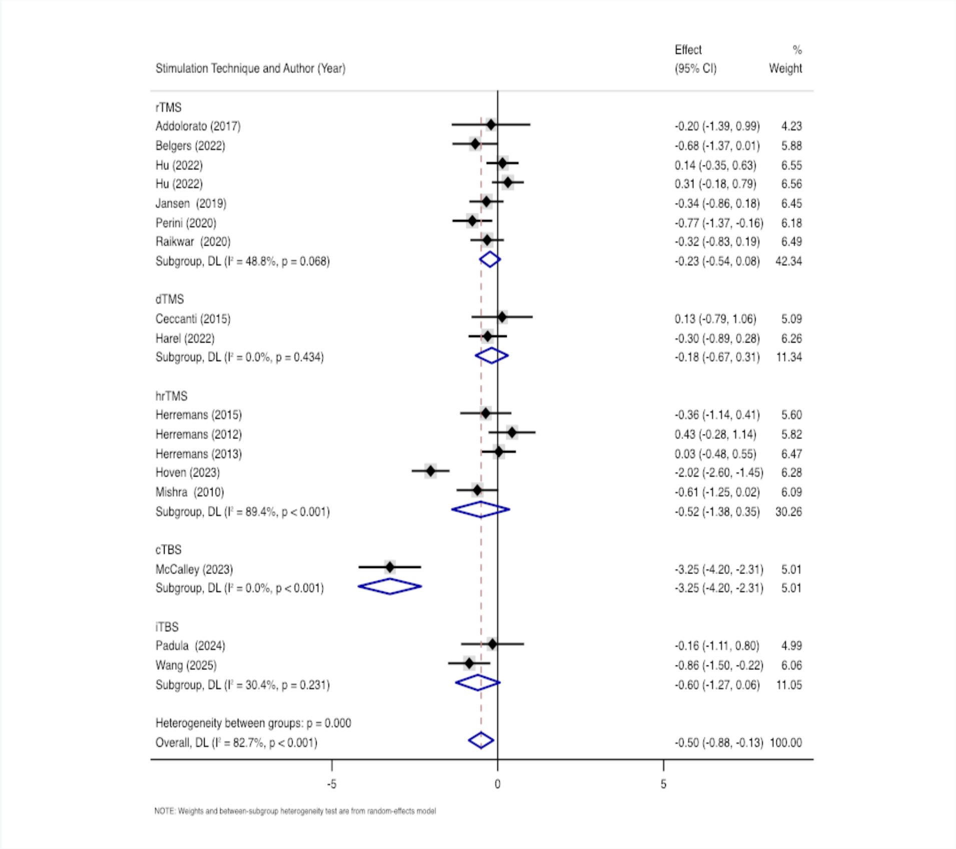


**Figure S2 Subgroup analysis based on stimulation mode**

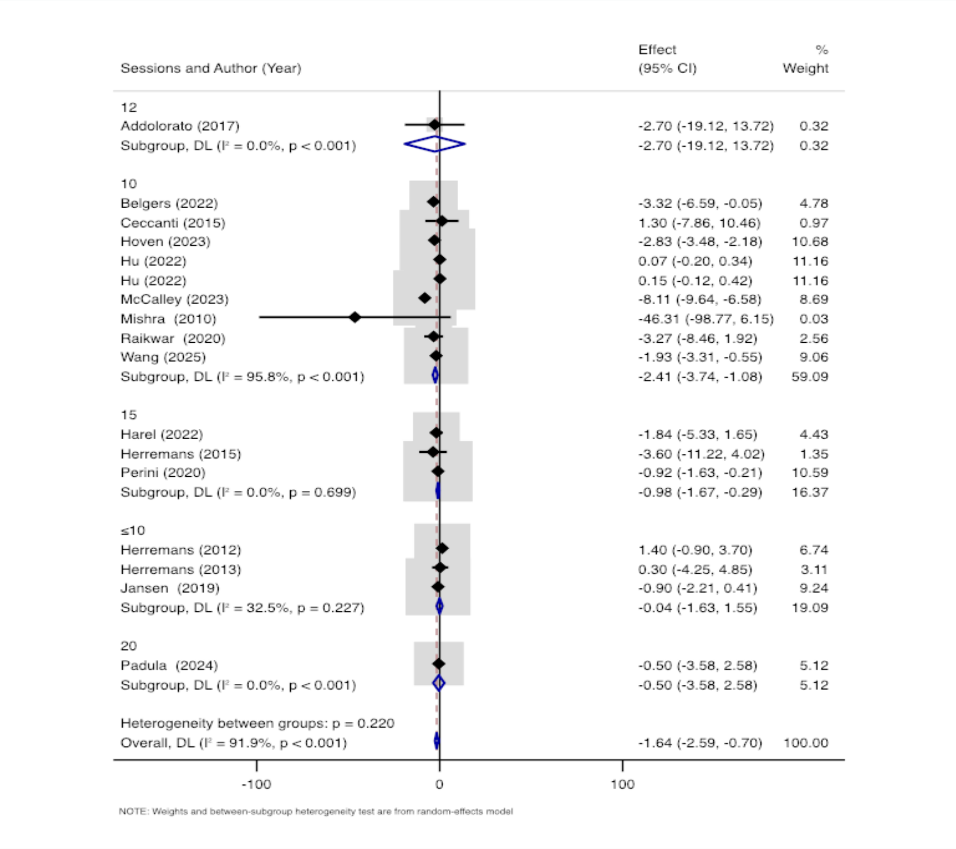


**Figure S3 Subgroup analysis based on stimulation sessions**

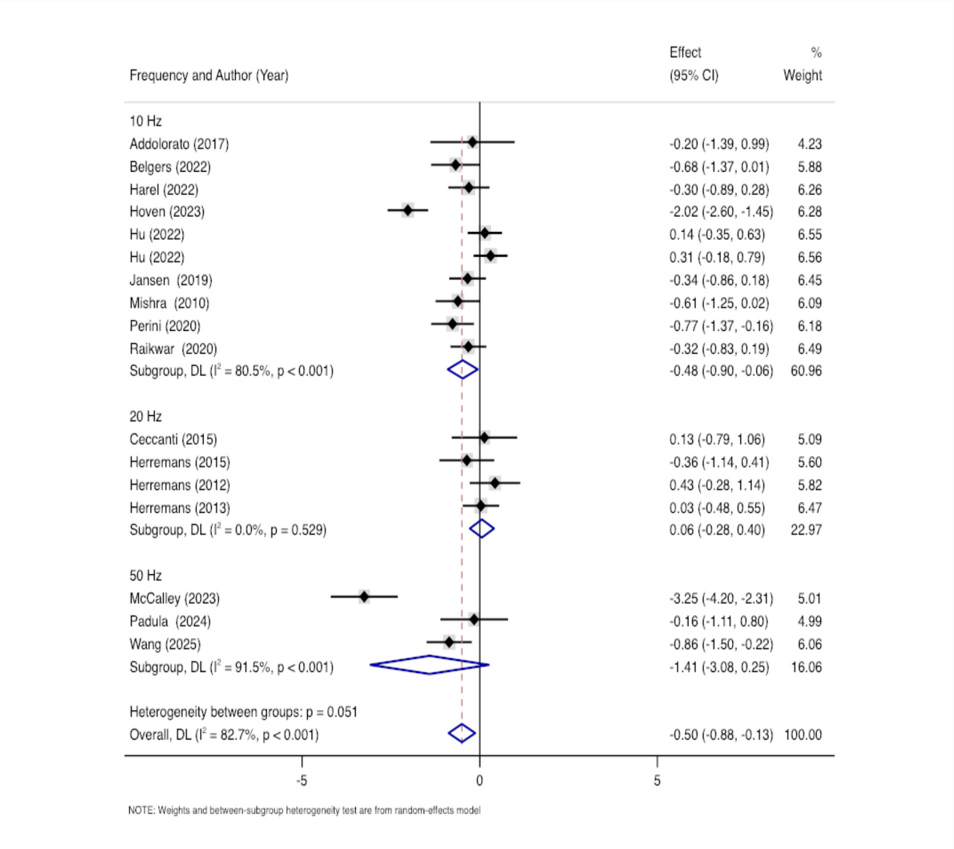


**Figure S4 Subgroup analysis based on stimulation frequency**

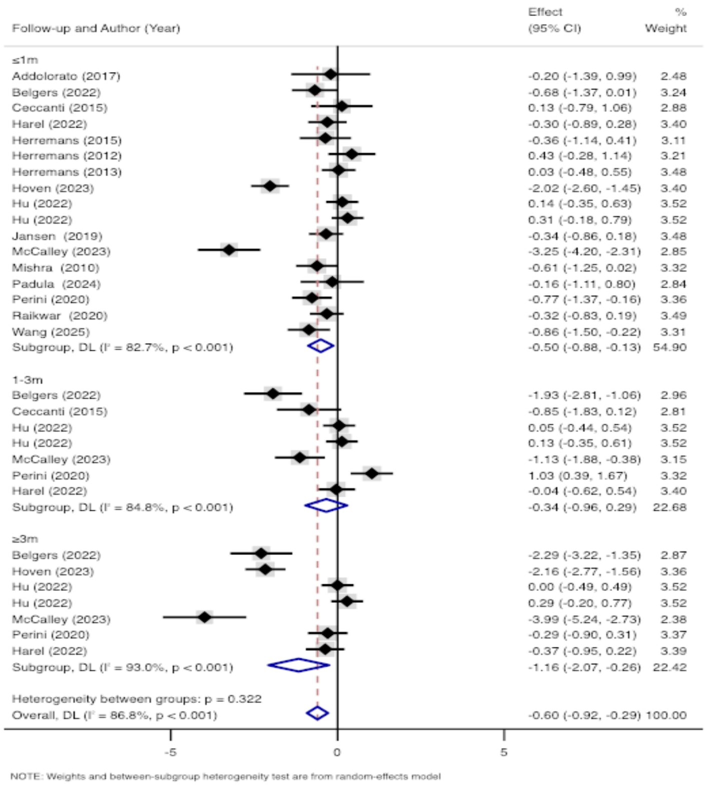


**Figure S5 Subgroup analysis based on follow-up duration**

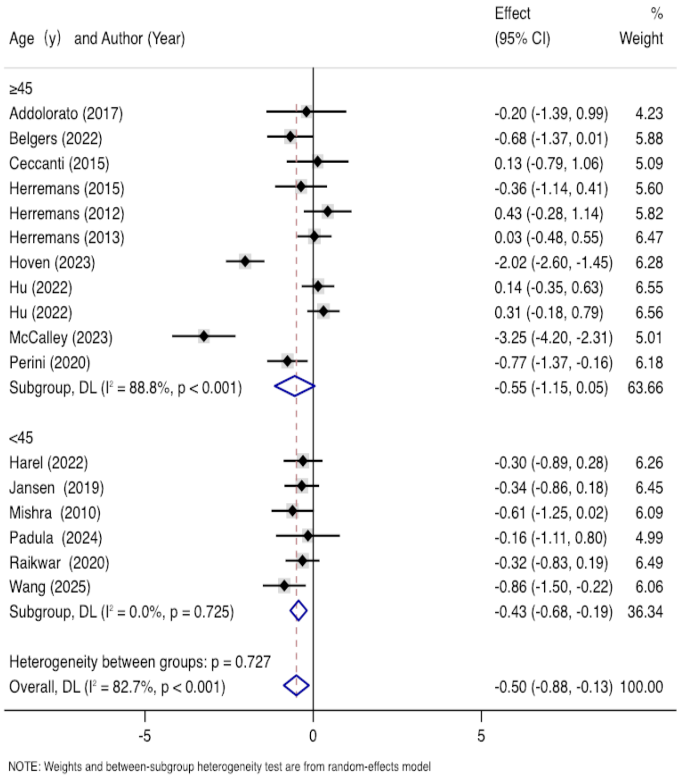


**Figure S6 Subgroup analysis based on age**

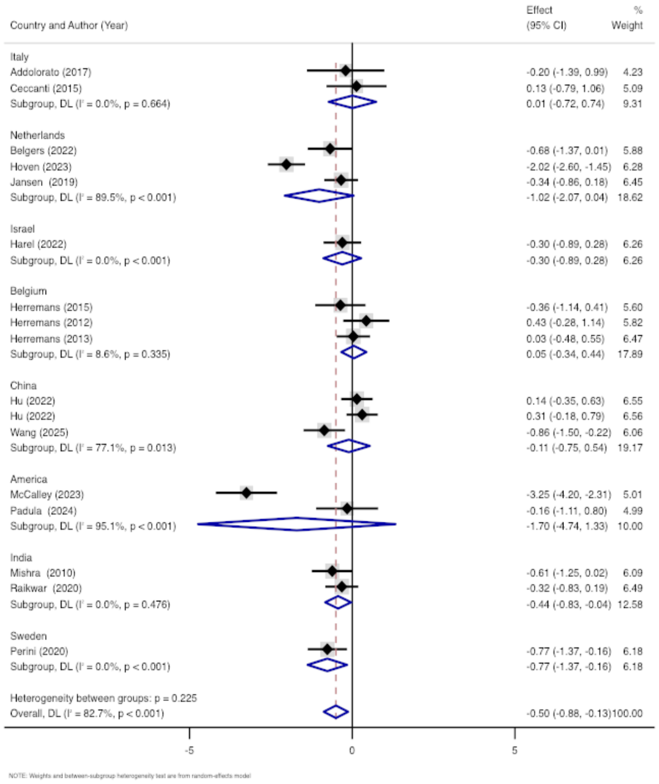


**Figure S7 Subgroup analysis based on country**

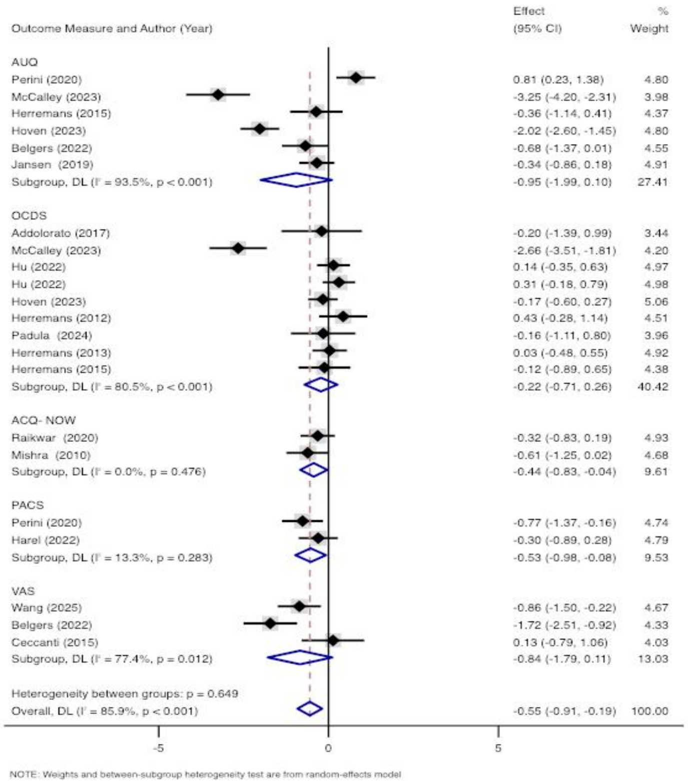


**Figure S8 Subgroup analysis based on assessment method**

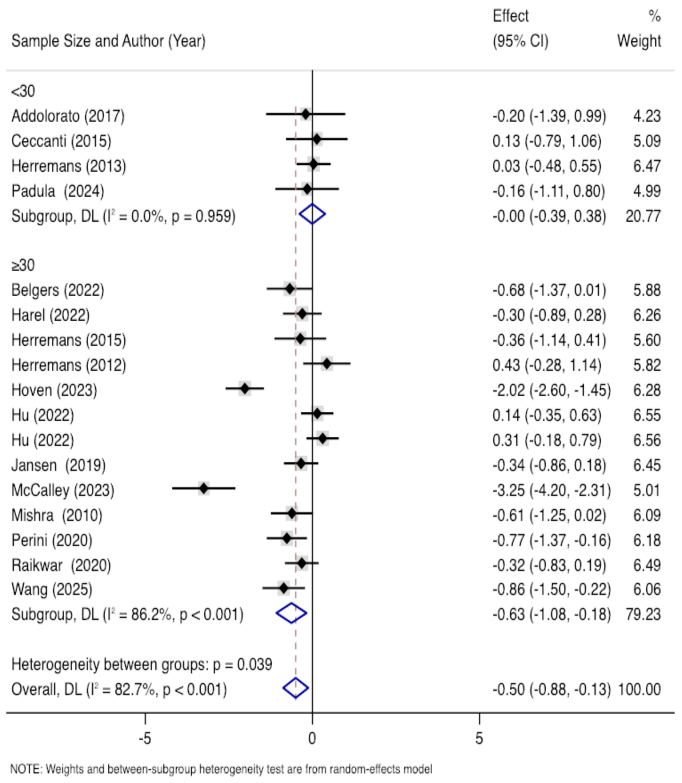


**Figure S9 Subgroup analysis based on sample size**

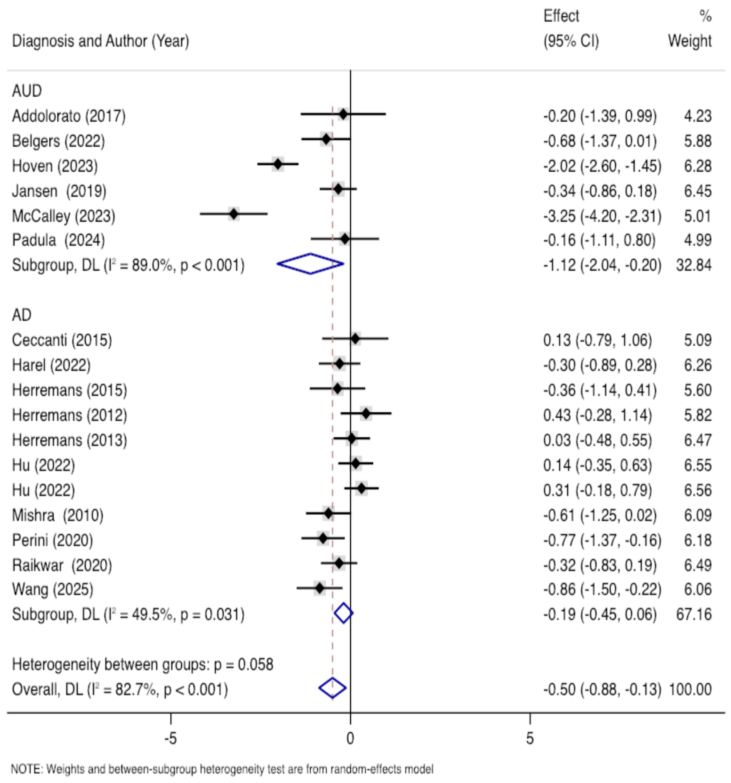


**Figure S10 Subgroup analysis based on disease type**

Meta-regression Number of obs = 17
**REML** estimate of between-study variance tau2 = .6557
% residual variation due to heterogeneity I-squared_res = 83.59%
Proportion of between-study variance explained Adj R-squared = -8.68%
**With** Knapp-Hartung modification

| _ES | exp(b) | Std. Err. | t | P>\|t\| | [95% Conf. | Interval] |
|---|---|---|---|---|---|---|
| stimulationregion | 1.013904 | .2478852 | 0.06 | 0.956 | .6021186 | 1.707307 |
| _cons | .581798 | .3914643 | -0.80 | 0.433 | .1386527 | 2.441272 |

**Figure S11 Meta regression based on stimulation site**

Meta-regression Number of obs = 17
**REML** estimate of between-study variance tau2 = .5516
% residual variation due to heterogeneity I-squared_res = 81.17%
Proportion of between-study variance explained Adj R-squared = 8.57%
**With** Knapp-Hartung modification

| _ES | exp(b) | Std. Err. | t | P>\|t\| | [95% Conf. | Interval] |
|---|---|---|---|---|---|---|
| stimulationtechnique | .8037119 | .1220494 | -1.44 | 0.171 | .5814733 | 1.11089 |
| _cons | 1.002271 | .4079955 | 0.01 | 0.996 | .4208903 | 2.386719 |

**Figure S12 Meta regression based on stimulation technique**

Meta-regression Number of obs = 17
**REML** estimate of between-study variance tau2 = 5.575
% residual variation due to heterogeneity I-squared_res = 92.23%
Proportion of between-study variance explained Adj R-squared = -8.48%
**With** Knapp-Hartung modification

| _ES | exp(b) | Std. Err. | t | P>\|t\| | [95% Conf. | Interval] |
|---|---|---|---|---|---|---|
| sessions | .9007562 | .5518766 | -0.17 | 0.867 | .2440407 | 3.324698 |
| _cons | .236657 | .3781731 | -0.90 | 0.381 | .0078507 | 7.133986 |

**Figure S13 Meta regression based on stimulation sessions**

Meta-regression Number of obs = 17
**REML** estimate of between-study variance tau2 = .6119
% residual variation due to heterogeneity I-squared_res = 83.28%
Proportion of between-study variance explained Adj R-squared = -1.43%
**With** Knapp-Hartung modification

| _ES | exp(b) | Std. Err. | t | P>\|t\| | [95% Conf. | Interval] |
|---|---|---|---|---|---|---|
| frequency | .7569163 | .2127847 | -0.99 | 0.338 | .4157394 | 1.37808 |
| _cons | .9311929 | .4531474 | -0.15 | 0.885 | .3300476 | 2.627258 |

**Figure S14 Meta regression based on stimulation frequency**

Meta-regression Number of obs = 17
**REML** estimate of between-study variance tau2 = .6562
% residual variation due to heterogeneity I-squared_res = 83.77%
Proportion of between-study variance explained Adj R-squared = -8.77%
**With** Knapp-Hartung modification

| _ES | exp(b) | Std. Err. | t | P>\|t\| | [95% Conf. | Interval] |
|---|---|---|---|---|---|---|
| Agey | .8981681 | .4104979 | -0.23 | 0.817 | .3390684 | 2.379184 |
| _cons | .7190971 | .5611672 | -0.42 | 0.679 | .1362731 | 3.794591 |

**Figure S15 Meta regression based on age**

Meta-regression Number of obs = 17
**REML** estimate of between-study variance tau2 = .6544
% residual variation due to heterogeneity I-squared_res = 83.75%
Proportion of between-study variance explained Adj R-squared = -8.46%
**With** Knapp-Hartung modification

| _ES | exp(b) | Std. Err. | t | P>\|t\| | [95% Conf. | Interval] |
|---|---|---|---|---|---|---|
| country | .9648055 | .0975647 | -0.35 | 0.728 | .7777355 | 1.196872 |
| _cons | .7048911 | .3466186 | -0.71 | 0.488 | .2471358 | 2.01052 |

**Figure S16 Meta regression based on country**

Meta-regression Number of obs = 17
REML estimate of between-study variance tau2 = .5911
% residual variation due to heterogeneity I-squared_res = 82.79%
Proportion of between-study variance explained Adj R-squared = 2.03%
With Knapp-Hartung modification

| _ES | exp(b) | Std. Err. | t | P>\|t\| | [95% Conf. | Interval] |
|---|---|---|---|---|---|---|
| samplesize | .5534826 | .2855297 | -1.15 | 0.269 | .1843178 | 1.662037 |
| _cons | 1.740122 | 1.647385 | 0.59 | 0.567 | .2313319 | 13.08953 |

**Figure S17 Meta regression based on sample size**

Meta-regression Number of obs = 17
REML estimate of between-study variance tau2 = .4184
% residual variation due to heterogeneity I-squared_res = 76.95%
Proportion of between-study variance explained Adj R-squared = 30.65%
With Knapp-Hartung modification

| _ES | exp(b) | Std. Err. | t | P>\|t\| | [95% Conf. | Interval] |
|---|---|---|---|---|---|---|
| diagnosis | 2.517463 | 1.001629 | 2.32 | 0.035 | 1.078107 | 5.878471 |
| _cons | .1293327 | .0894732 | -2.96 | 0.010 | .0296019 | .5650635 |

**Figure S18 Meta regression based on disease type**

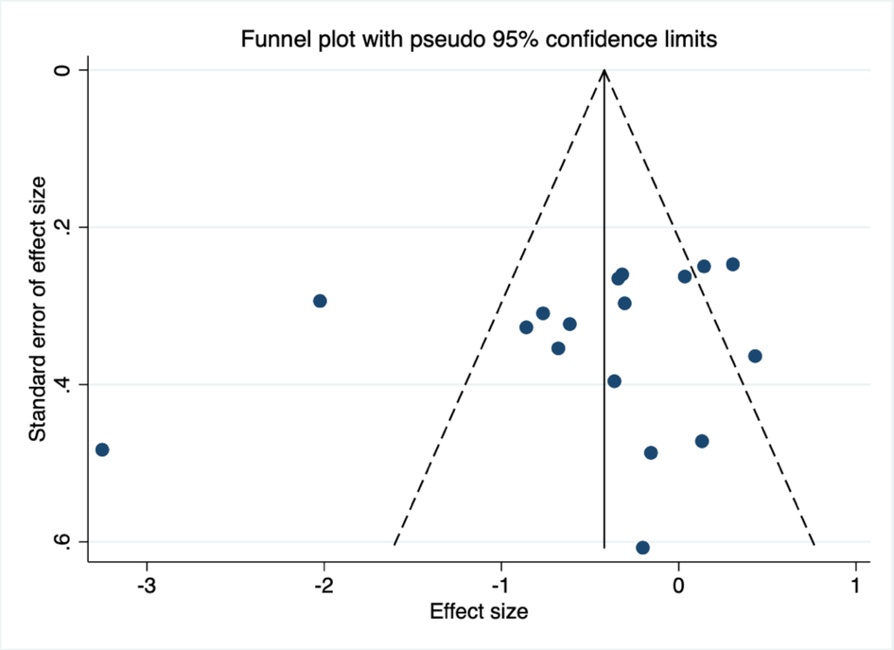


**Figure S19 The funnel plot of alcohol craving**

Number of studies = 17 Root MSE = 2.385

| Std_Eff | Coef. | Std. Err. | t | P>\|t\| | [95% Conf. | Interval] |
|---|---|---|---|---|---|---|
| slope | .4401895 | .7908828 | 0.56 | 0.586 | -1.245537 | 2.125916 |
| bias | -2.76392 | 2.470484 | -1.12 | 0.281 | -8.029633 | 2.501793 |

Test of H0: no small-study effects P = 0.281

**Figure S20 Egger's test for alcohol craving**

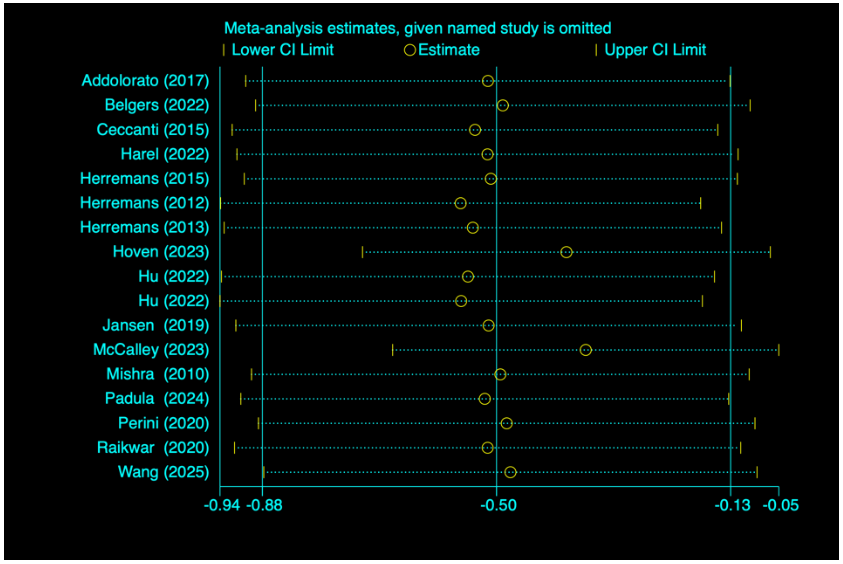


Figure S21 Sensitivity analysis of alcohol craving

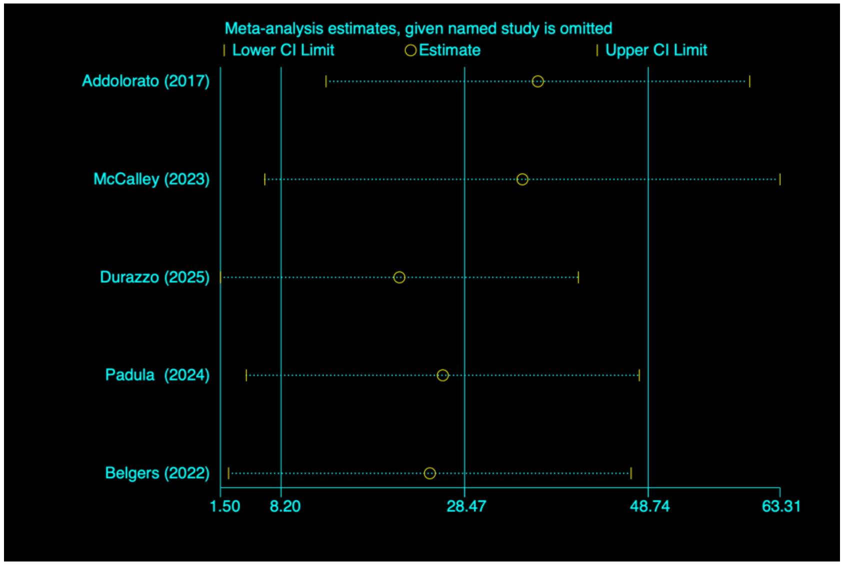


Figure S22 Sensitivity analysis of abstinence days

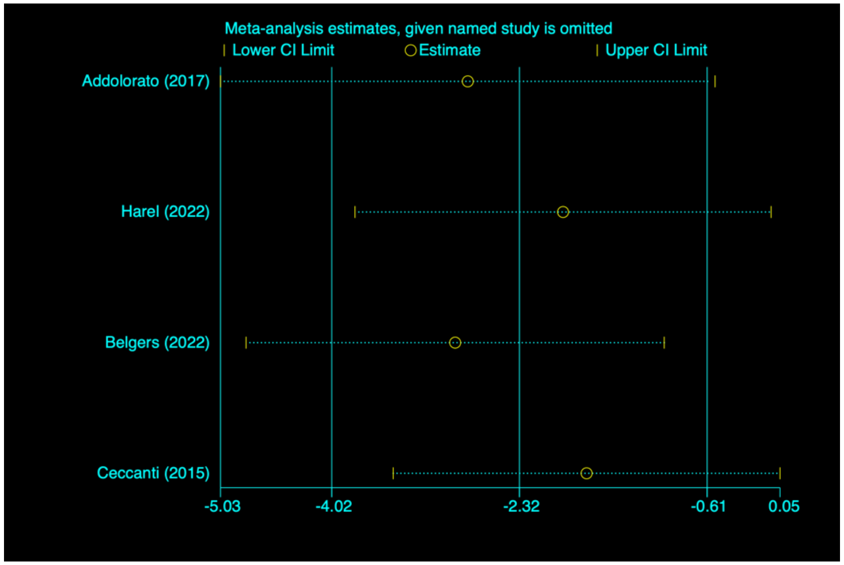


Figure S23 Sensitivity analysis of alcohol intake

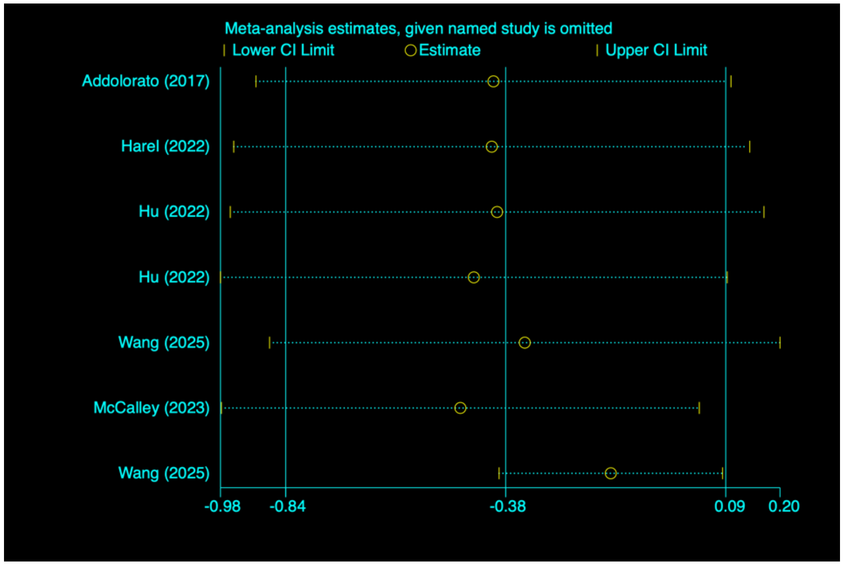


Figure S24 Sensitivity analysis of anxiety

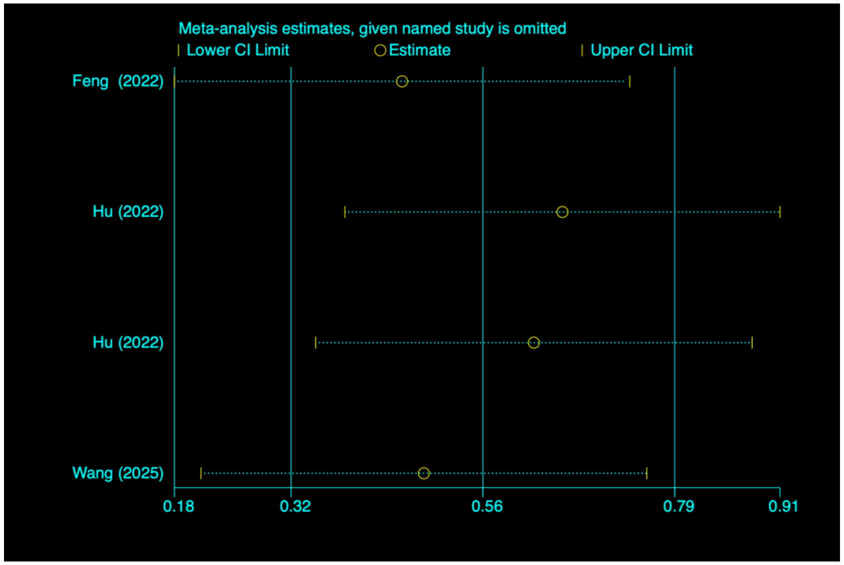


**Figure S25 Sensitivity analysis of depression**

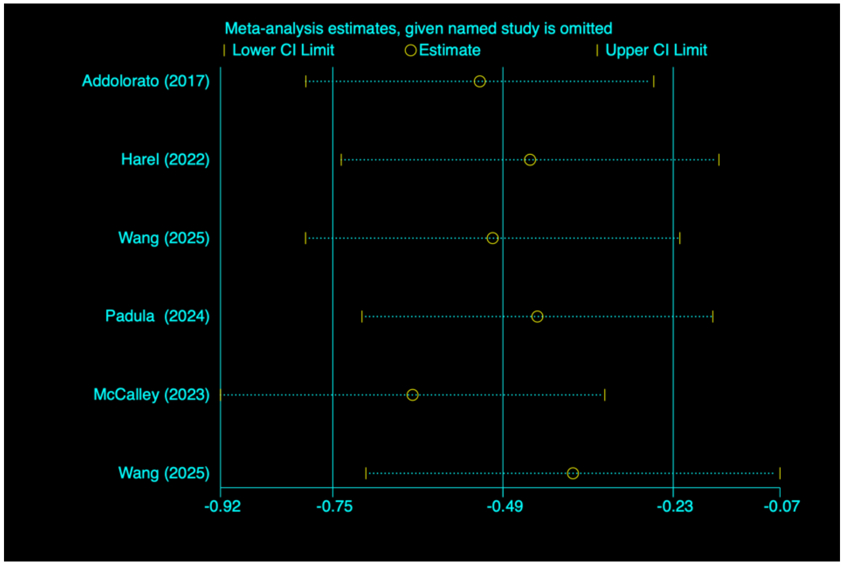


**Figure S26 Sensitivity analysis of cognitive function**
